# Chemical Space Charting of Different Parts of *Inula nervosa* Wall.: Upregulation of Expression of Nrf2 and Correlated Antioxidants Enzymes

**DOI:** 10.3390/molecules25204789

**Published:** 2020-10-19

**Authors:** Xiang-rong Cheng, Wei Zhao, Wen-le Dong, Guo-wei Le

**Affiliations:** 1 School of Food Science and Technology, Jiangnan University, Wuxi 214122, China; zhaowei1997w@163.com (W.Z.); dddddddddddd1212@163.com (W.-l.D.); lgw@jiangnan.edu.cn (G.-w.L.); 2 National Engineering Research Center for Functional Food, Jiangnan University, Wuxi 214122, China; 3 Collaborative Innovation Center of Food Safety and Quality Control in Jiangsu Province, Jiangnan University, Wuxi 214122, China

**Keywords:** *Xiaoheiyao*, GNPS molecular networking, MolNetEnhancer, XCMS online, Nrf2

## Abstract

The edible and medicinal part of *Inula nervosa* Wall. (*Xiaoheiyao*) is confined to its root without sufficient phytochemical and biological investigation. In this study, the secondary metabolites of root, stem, leaf, and flower of *I. nervosa* Wall. were visualized using Global Natural Products Social Molecular Networking (GNPS), MolNetEnhancer, XCMS(xcmsonline.scripps.edu) analysis, and `ili mapping based on high performance liquid chromatography-tandem mass spectrometry (HPLC-MS/MS) data to reveal their chemical differences. Among the 11 kinds of chemical repertoires annotated by MolNetEnhancer and 16 hits against the GNPS library, 10-isobutyryloxy-8,9-epoxythymol isobutyrate (**1**) was revealed as the most dominant and responsible marker between the roots and the other parts. Moreover, a battery of unique MS features as well as differential markers were discovered from different parts of the plant. The chemical differences contribute to the bioactivity differences, which presented in the 2,2-diphenyl-1-picryl-hydrazyl (DPPH)assay and H_2_O_2_-insulted HepG2 cells and were in significant correlations with the contents of **1**. real-time reverse transcription polymerase chain reaction (RT-PCR)results demonstrated that *I. nervosa* Wall. extracts upregulated the mRNA expression of nuclear factor E2-related factor 2(Nrf2), heme oxygenase 1(HO-1), NAD(P)H quinone dehydrogenase 1 (NQO1), manganese superoxide dismutase (MnSOD), and glutamate-cysteine ligase catalytic subunit (GCLC) actors involved in antioxidative response in H_2_O_2_-challenged HepG2 cells. These findings support the roots of *I. nervosa* Wall. as active parts of *Xiaoheiyao*, and also indicate the potential antioxidant activities of other parts.

## 1. Introduction

*Inula nervosa* Wall. is an edible and medicinal herb distributed throughout Southwest of China [1]. The roots of *I. nervosa* Wall. (*Xiaoheiyao*) are traditionally applied as culinary spice and folk medicine for treating stomachache and relieving rheumatism [2]. Phytochemical investigation and bioactivity assay on *I. nervosa* Wall. have mainly revealed thymols, phenylpropanoids, and diterpenes with nitric oxide and melanogenesis inhibitory activities [2,3,4], which dramatically differ from batch of electrophilic sesquiterpene lactones discovered from congeneric *Inula* species [5,6,7]. Furthermore, only the root of *I. nervosa* Wall. was approved as a new food material by the Ministry of Health of PR China in 2010 [8], whereas there is limited scientific information on the other parts and their applications.

Electrophilic natural products are the principal indirect antioxidants in culinary spices, fruits and vegetables, which are responsible for the activation of the kelch-like ECH-associated protein-1/nuclear factor E2-related factor 2/antioxidant response element(Keap1/Nrf2/ARE) signaling pathway, resulting in transcriptional induction of diverse cytoprotective phase 2 enzymes, including glutathione transferases (GSTs), heme oxygenase 1 (HO-1), UDP-glucuronosyltransferases (UGTs), and NAD(P)H:quinone oxidoreductase 1 (NQO1) [9,10,11]. In distinction with direct antioxidants, the indirect antioxidants are not consumed, with long half-lives, and are more robust to counteract the damaging effects of oxidants and electrophiles which play a role in the pathogenesis of chronic diseases, such as cancer, neurodegeneration, atherosclerosis, and non-alcoholic fatty liver disease [9]. Hence, the development of electrophilic natural products as promising antioxidants is gaining attention from pharmacologists and nutritionists.

The recently launched Global Natural Product Social (GNPS) molecular networking is an open-access knowledge base for community-wide organization and sharing of raw, processed, or identified tandem mass (MS/MS) spectrometry data [12]. Molecular networking groups MS/MS spectra based on spectral similarity, which also facilitate to dereplicate known molecules by spectral comparisons with trusted standards or community annotations, resulting in an information-rich plot of the mass spectrometry detectable chemical space [12]. In this study, the chemical space of secondary metabolites from root, stem, leaf, and flower parts of *I. nervosa* Wall. was visualized and annotated by GNPS molecular networking, MolNetEnhancer, XCMS((xcmsonline.scripps.edu) analysis, and `ili mapping based on high performance liquid chromatography-tandem mass spectrometry (HPLC-MS/MS) analysis. Moreover, the antioxidant activities of different parts of *I. nervosa* Wall. were evaluated on the 2,2-diphenyl-1-picryl-hydrazyl (DPPH) scavenging assay and hydroperoxide (H_2_O_2_)-induced HepG2 cells for addressing the antioxidant difference.

## 2. Materials and Methods

### 2.1. Materials

Six batches of the whole plants of *I. nervosa* Wall. were collected from Liangwang Mountain, Kunming, Yunnang Province, PR China, in September 2018, and identified by Prof. Guo-Dong Li (Kunming Zhifen Biotechnology Co., Ltd.). The voucher specimens (No. XHY20180901–XHY20180906) are deposited at School of Food Science and Technology, Jiangnan University, Wuxi, Jiangsu Province, PR China. The human hepatoma carcinoma cell lines (HepG2) were obtained from the Cell Bank of the Chinese Academy of Sciences (Shanghai, China).

### 2.2. Chemicals and Primary Detection Kits

Dulbecco’s modified Eagle’s medium (DMEM/High glucose) was purchased from Hyclone (Logan, UT, USA), 0.25% trypsin solution with ethylene diamine tetraacetic acid(EDTA), fetal bovine serum (FBS), and penicillin streptomycin were purchased from Gibco (Grand Island, NY, USA). Detection kits of total antioxidant capacity (T-AOC), thiobarbituric acid reactive substances (TBARS), glutathione peroxidase (GSH-PX), and catalase (CAT) were purchased from Nanjing Jiancheng Bioengineering Institute (Nanjing, China). Assay kits of reaction oxygen species (ROS), cell counting kit-8 (CCK-8), and enhanced bicinchonininc acid (BCA) protein were purchased from Beyotime Biotechnology (Shanghai, China). RNA-easy Isolation Reagent kit was purchased from Vazyme (Nanjing, China). PrimeScript RT (reverse transcription) Master Mix reverse transcription kit, SYBR green PCR kit, and the q225 were purchased from Monad (Suzhou, China). DPPH, quercetin, and HPLC-grade solvents were purchased from Shanghai Aladdin Bio-Chem Technology Co., Ltd. (Shanghai, China). HPLC-grade water was obtained by filtration using a Milli-Q Direct water purification system from Millipore (Billerica, MA, USA) unless otherwise stated.

### 2.3. Crude Extract Preparation of Different Parts of I. nervosa Wall

The air-dried and powdered root, stem, leaf, and flower parts of *I. nervosa* Wall. (each 10 g) were extracted with ethanol (1:20) three times with ultrasonic assistant for 30 min at 45 °C. The combined extracts were concentrated under reduced pressure to afford crude extracts of different parts of *I. nervosa* Wall. (IEs), which were further dissolved in methanol at 1.0 mg/mL and filtered by 0.2 µm membrane.

### 2.4. HPLC-MS/MS Analysis

The HPLC-MS/MS analyses were developed on an HPLC system (Agilent 1200) coupled to a Thermo-Finnigan LCQ Advantage ion-trap mass spectrometer (San Jose, CA, USA) fitted with an electrospray ionization source operating in positive mode. The chromatographic separation was carried out on a Kinetex C_18_ column (Phenomenex; 150 mm × 4.6 mm × 5 µm), using an isocratic 10% acetonitrile (ACN) for 2 min and a gradient from 10% to 99% ACN over 20 min followed by 4 min washing and 4 min equilibrium with a flow rate of 0.6 mL/min. Both solvents contained 0.1% formic acid. The HPLC eluate was electrospray ionized at 35 eV with capillary temperature 325 °C. An untargeted method was employed to acquire the MS/MS spectra within *m/z* range 100–2000.

### 2.5. Molecular Networking Creation and Annotation

A molecular network comprising MS/MS data of IEs was created using the online workflow at the GNPS website [12]. The MS/MS data were clustered with a precursor and fragment ion mass tolerance of 2.0 and 0.5 Da respectively, to create consensus spectra. The consensus spectra containing less than 2 spectra were discarded. A network was then built where edges were filtered to have a cosine score above 0.7 and more than 6 matched peaks. The consensus spectra in the network were dereplicated against spectra in GNPS’ libraries which were filtered in the same threshold as the input data. Analogue search was enabled against the library with a maximum mass shift of 100 Da. The achieved GNPS molecular networking was further annotated with MolNetEnhancer [13]. The generated molecular network was imported into Cytoscape v3.7.1 (The Cytoscape Consortium, New York, NY, USA) and displayed as nodes and edges [14]. Nodes represent parent ions, and edge thickness corresponds to the cosine score between two nodes.

### 2.6. Multivariate Analyses

HPLC-MS data, including the retention time and corresponding parent mass spectra, were submitted to XCMS Online platform which was developed to process and visualize mass-spectrometry-based, complex untargeted metabolomic data [15]. For feature detection, the ‘matchedFilter’ method was applied, with a maximal tolerated *m*/*z* deviation in consecutive scans of 50 ppm, minimum peak width in 10 s, maximum peak width in 60 s, signal/noise threshold as 6, and minimum *m*/*z* difference as 0.01. The ‘obiwarp’ method was adopted for retention time correction with step size of 1 *m*/*z* for profile generation from the raw data files. The peaks were aligned according to bandwidth threshold as 15 s, minimum fraction of samples as 0.5, and width of overlapping *m*/*z* slices as 0.05. The parameters for statistics and visualization were set as default.

### 2.7. `ili Visualization

A metadata file comprising intensity of inspected MS feature and coordinates was created and saved in comma-separated values(CSV) format. The spatial data mapping was achieved by uploading the photo of *I. nervosa* Wall. and metadata file [16]. A logarithmic scale was set to visualize the intensity of targeted feature in different parts of *I. nervosa* Wall.

### 2.8. DPPH Radical Scavenging Activity Assay

DPPH radical scavenging activity was evaluated by spectrophotometric analysis (Microplate Reader, Biotek, Winooski, VT, USA), as previously reported [17]. In brief, 2 mL DPPH solutions (0.1 M) were mixed with 1 mL samples in gradient concentrations (0.125, 0.25, 0.5, 1.0, 2.0 mg/mL) and kept at room temperature for 30 min, then optical density values were measured at 517 nm.

### 2.9. Intracellular Antioxidative Activity Assay

As previously reported, the IEs were evaluated for intracellular antioxidative activities on H_2_O_2_-induced HepG2 cells [18]. Cells were maintained in DMEM supplemented with 10% FBS, 100 U/mL penicillin, and 100 µg/mL streptomycin, in an incubator with 37 °C and humidified 5% CO_2_ atmosphere. Cell viabilities after treating with gradient concentrations of IEs and H_2_O_2_ were determined using a cell counting kit-8 (CCK-8) assay. HepG2 cells were treated with H_2_O_2_ at 0.1–1.6 mM for 4 h and a suitable concentration was determined to induce intracellular oxidative stress. Cells were pre-cultured with IEs at 0.5, 1.0, and 2.0 mg/mL for 24 h, then renewed with DMEM and treated with H_2_O_2_ for another 4 h.

Intracellular ROS levels were measured using a 2′,7′-dichlorodihydrofluorescein diacetate (DCFH-DA) probe according to the kit instruction. Cells were washed with PBS and co-incubated with DCFH-DA for 30 min, then observed under a fluorescence microscope (Zeiss, Jena, Germany) and fluorescence intensity was measured under a microplate reader (Biotek, Winooski, VT, USA) (λ_excitation_ = 488 nm, λ_emission_ = 525 nm).

The levels of TBARS, T-AOC, CAT, and GSH-PX were measured according to the kits’ instructions after cells were lysed with phenylmethylsulfonyl fluoride (PMSF) dissolved radio immunoprecipitation assay (RIPA) lysis buffer in ice for 10 min and centrifuged at 12,000× *g* for 5 min at 4 °C to afford the supernatant [19].

### 2.10. Real-Time Reverse Transcription Polymerase Chain Reaction (RT-PCR)

Total RNA was isolated from HepG2 cells using the RNA-easy Isolation Reagent kit and reverse-transcribed into cDNA using the PrimeScript RT Master Mix reverse transcription kit. The resultant cDNA was amplified by PCR using the SYBR green PCR kit and then the expression was quantified by the q225. Primers for RT-PCR are shown in Table 1. Gene expression was calculated using the 2^−ΔΔCt^ method and normalized to β-actin.

### 2.11. Statistical Analysis

All data were expressed as means ± S.E.M. (standard error of mean), obtained from at least six separate repetitions, and one representative image was selected for presentation in the Figures. Statistical analysis was performed using IBM-SPSS Inc. software (version 20.0, Armonk, NY, USA). One-way analysis of variance (ANOVA) was used to determine significant differences between means, with the significance level taken at *p* < 0.05. Tukey’s test was used to perform multiple comparisons between means, with the significance level *p* < 0.05.

## 3. Results and Discussion

### 3.1. Molecular Networking and Annotation of IEs

**Figure 1:**
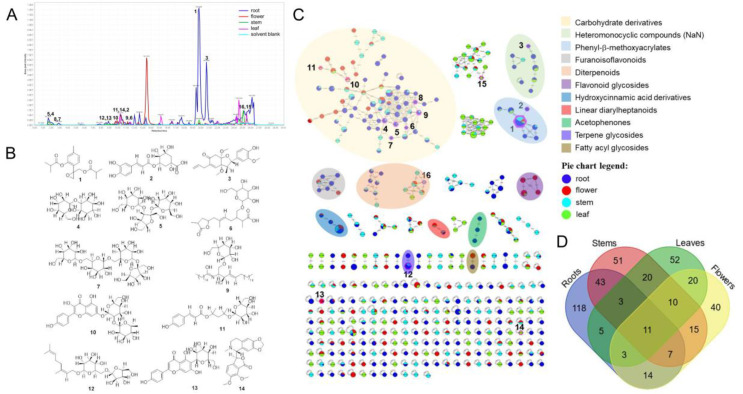
Chemical characterization of secondary metabolites from IEs basing on high performance liquid chromatography-tandem mass spectrometry (HPLC-MS/MS). (**A**) base peak chromatogram (BPC) of representative IE samples in positive mode. (**B**) Several chemical structures of the hits in tolerance against GNPS spectral library. (**C**) The molecular network of IEs [20], in which chemical classification was achieved by MolNetEnhancer at the subclass level, except for the heteromonocyclic compounds classified at the molecular framework level [21] In the molecular network, nodes represent parent ions and their sizes were designed according to the sum of precursor intensity. Nodes from solvent blanks were removed. Pie ratio was determined according to scan number of spectra. Edges represent the degree of similarity between the connected nodes and their thicknesses were corresponding to cosine scores. (**D**) Venn diagram with the precursor ions presented in the molecular network.

Molecular networking is a visualization approach of the chemical space present in tandem MS experiments [12]. The HPLC-MS/MS spectra of IEs were grouped and constructed to create a molecular network based on spectral similarity (Figure 1). The molecular network exhibits an overview of the secondary metabolites and their distributions among the samples. After solvent blank removal, 412 nodes organized in 244 clusters were exhibited in the molecular network (Figure 1C). For all that, 118 parent ions were detected exclusively in root samples, 51 parent ions were found exclusively in stem samples, 52 parent ions were discovered exclusively in leaf samples, 40 parent ions were detected exclusively in flower samples, and only 11 features were shared by all of them (Figure 1D, Supporting Information Tables S1–S4). The chemical diversity of different parts of *I. nervosa* Wall. distinguished in the base peak chromatograms (BPCs) as well (Figure 1A), with remarkable differences of MS feature and intensity among root, stem, leaf, and flower extracts.

MolNetEnhancer is a workflow that combines the outputs from various in silico annotation tools and classifies chemicals through ClassyFire, which could provide a comprehensive chemical overview of metabolomic data [13,22]. As shown in Figure 1C, the molecular families were classified at the subclass level, except for the heteromonocyclic compounds. The group of heteromonocyclic family was classified at the molecular framework level since NaN (not-a-number) for these nodes was retrieved at the subclass level. The chemical interpretation of secondary metabolites from IEs reveals mainly 11 types of structures, including carbohydrate derivatives, heteromonocyclic compounds, phenyl-β-methoxyacrylates, furanoisoflavonoids, diterpenoids, flavonoid glycosides, hydroxycinnamic acid derivatives, linear diarylheptanoids, acetophenones, terpene glycosides, and fatty acyl glycosides.

The online dereplication against the GNPS library, the most comprehensive spectral library of natural products [23], resulted in 183 hits, which were further manually checked for the mirror spectra, cosine scores, and number of shared peaks, to yield 16 hits in tolerance (Table 2) and 54 homologues/derivatives (Supplementary Table S5). The 16 hit compounds were empirically classified as saccharides (**4**, **5**, **7**, **8**), terpenoids (**1**, **6**, **12**), phenylpropanoids (**2**, **11**), flavonoids (**10**, **13**), chlorophylls (**15**, **16**), and others (**3**, **9**, **14**), which are consistent with the molecular families annotated by MolNetEnhancer. Although some thymols, phenylpropanoids, and diterpenes have already been reported [2,3,4], all of these hit compounds were discovered from *I. nervosa* Wall. for the first time. Meanwhile, the vast majority of nodes evaded all attempts to interpret them, indicating that there exists plenty of dark matter of metabolites from IEs. It is noteworthy that 10-isobutyryloxy-8,9-epoxythymol isobutyrate (**1**) shows as the strongest MS signal in the BPC and the biggest node in the molecular network, indicating it as the major component in the roots. Moreover, compounds **3**, **6**, **7**, and **8** were discovered exclusively in the roots, compound **16** was discovered exclusively in the stems, and compounds **14** and **15** were discovered exclusively in the leaves. Some of these compositions have phytobiological effects, such as chlorophyll helping plants’ photosynthesis and resveratrol protecting against pests and diseases.

### 3.2. Multivariate Analyses of IEs

**Figure 2:**
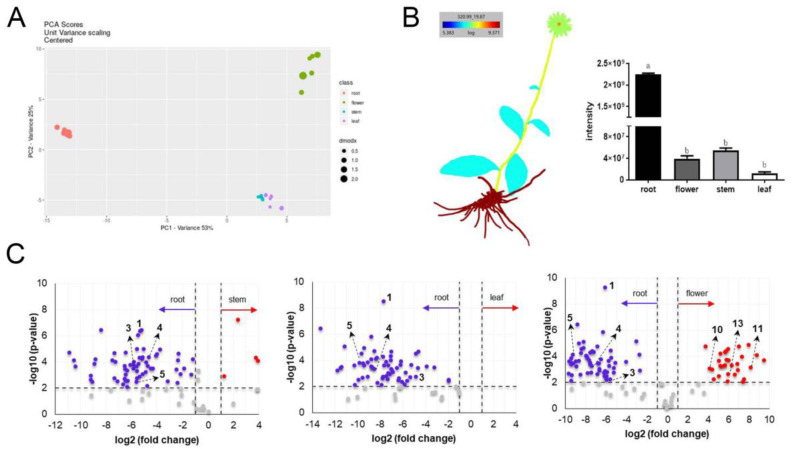
Multivariate analyses and visualization of metabolites from IEs. (**A**) PCA (Principal Component Analysis) plot of IEs based on parent ion intensity. (**B**) `ili visualization of the significant feature (compound **1**, *m*/*z* 320.9909) intensity at the logarithmic scale (*p* < 0.05 between root and other parts, *p* = 0.931 between flower and stem, *p* = 726 between flower and leaf, *p* = 0.409 between stem and leaf). (**C**) Volcano plots for significant metabolite markers comparing between root with stem, leaf, and flower of *I. nervosa* Wall. (fold change > 2, *p* < 0.01).

In order to obtain more detailed information about the metabolic profiling of IEs, the HPLC-MS data were submitted to the XCMS Online platform for a multigroup comparative analysis. Principal component analysis (PCA) conducted at the unit variance scale showed the distinction of roots with other parts and the similarity of stems with leaves in the profiling of secondary metabolites (Figure 2A). A batch of metabolites were responsible for the significant distinctions (Supplementary Table S6), in which compound **1** (*m*/*z* 320.9909) was found to be the most responsible marker (*p* < 0.01, *q* < 0.01, CV < 0.01). The concentration difference of **1** in IEs was further mapped by `ili based on the MS intensity (Figure 2B), showing an exponential difference in the concentration of **1** with a sequence of root > stem > flower > leaf.

Pairwise analyses between root with other parts were further performed to reveal their chemical differences. The significant markers were exhibited on volcano plots (fold change > 2, *p* < 0.01) (Figure 2C, Supplementary Tables S7–S9). A number of scatters were observed on the left of volcano plots, which represent the dominant features from roots, whereas much fewer dominant features (on the right) were observed from stems and leaves. Comparing the IEs of root with flower, both of them contained plenty of dominant metabolites, suggesting their great difference in secondary metabolites. Among the significant markers, compounds **1**, **3**, **4**, and **5** dominated in root and compounds **10**, **11**, and **13** dominated in flower were identified via the aforementioned molecular networking. However, most of the markers are still obscure and need further chemical annotation.

The previous study on ethanol extracts of the aerial parts and underground parts of *I. nervosa* Wall. have revealed higher contents of total polyphenols and total flavonoids and stronger antioxidative activities from the underground parts than the aerial parts [24]. The root parts have also been reported with high content of bioactive thymol and thymyl isobutyrate [25]. Compound **1** discovered as a dominant marker in this study is a thymol derivative featured an epoxy moiety. The ring-strained epoxide is prone to undergo a Michael addition with nucleophilic sites, such as cysteine, serine, lysine residues in proteins, DNA, and glutathione, interfering with a variety of biological functions [11]. Therefore, compound **1** might play a role in the bioactivity and traditional application of *Xiaoheiyao*. Meanwhile, as supporting from the chemical markers **3**, **10**, **11**, and **13**, the chemical constituents of polyphenols and flavonoids from IEs may vary in different parts. Moreover, for a comprehensive chemical comparison between different parts of *I. nervosa* Wall., the residue markers, such as the significant feature of *m*/*z* 787.2664 at 13.56 min (Figure 1A and Figure 2C), deserve further chemical identification.

### 3.3. DPPH Radical Scavenging Activity

Dietary antioxidants conduct a one-electron reaction with free radicals in vitro. DPPH has been widely applied for the determination of in vitro antioxidant activity of plant extracts and pure compounds due to its relatively stability.

**Figure 3:**
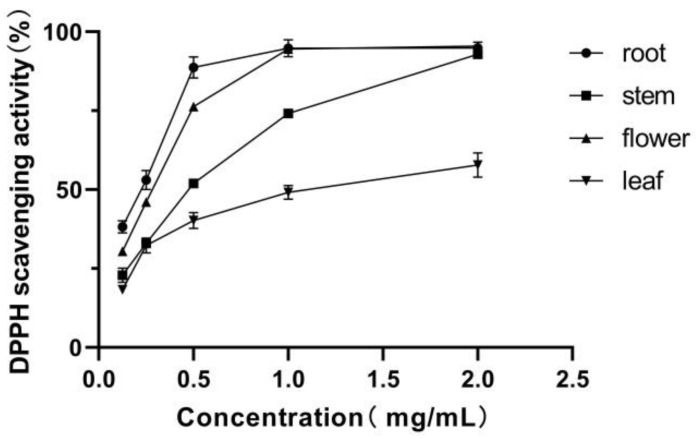
DPPH radical scavenging activities of IEs. Each value is expressed as means ± S.E.M. (*n* = 6).

The DPPH scavenging activities of IEs were evaluated and compared in Figure 3. The IEs scavenged the DPPH radical in a dose-dependent manner at concentrations of 0.125–2.0 mg/mL, and DPPH radicals were almost quenched by the root and flower IEs at 1.0 mg/mL. Quercetin with an half maximal inhibitory concentration (IC_50_) value of 3.47 ± 0.10 µg/mL was applied as a positive control. The root and flower IEs exhibited similar IC_50_ values of DPPH scavenging activity at 0.218 ± 0.04 and 0.284 ± 0.01 mg/mL, whereas the stem and leaf IEs owned IC_50_ values at 0.479 ± 0.03 and 1.055 ± 0.05 mg/mL, respectively. The DPPH scavenging activities of IEs are in descending order as root > flower > stem > leaf. The extracts from underground parts of *I. nervosa* Wall. have stronger radical scavenging activities than the aerial parts, which might owe to higher content of total polyphenols and total flavonoids, as in a previous report [24].

### 3.4. Intracellular Antioxidative Activities of IEs

Hydroperoxide is one of the most abundantly endogenic oxidizers inducing intracellular oxidative stress. Enzymatic removal of non-radical electrophiles in two-electron redox reactions, such as CAT and GSH-PX, neutralizing H_2_O_2_ into water and oxygen, is considered as the major intracellular anti-oxidative mechanism [26,27].

**Figure 4:**
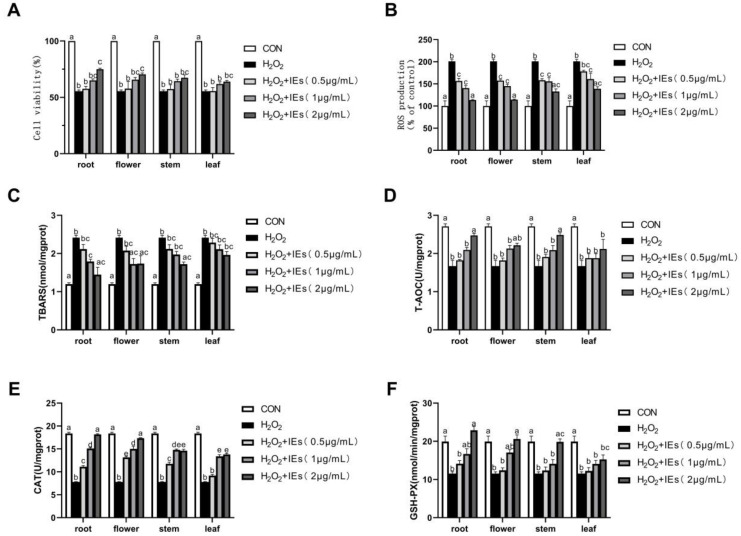
Effects of IEs on (**A**) cell viability, (**B**) ROS production, (**C**) TBARS level, (**D**) T-AOC level, (**E**) CAT, and (**F**) GSH-PX activities in H_2_O_2_-induced HepG2 cells. Values were presented as mean ± S.E.M. (*n* = 8). Bars within a figure without a common superscript differ significantly at *p* < 0.05.

In the present study, H_2_O_2_-induced HepG2 cells were used to evaluate the intracellular anti-oxidative activities of IEs. When the HepG2 cells were treated with 1.2 mM H_2_O_2_ for 4 h, the cell viability was decreased to 55.94% (*p* < 0.05) relative to control and ROS production increased to 200.97% (*p* < 0.05) relative to control. The intracellular total antioxidant capability and the enzyme activities to eliminate H_2_O_2_ was significantly lower than the control, while the TBARS level was a fold increase over the control (Figure 4).

The IEs were initially confirmed with no significant cytotoxic effect on HepG2 cells at the inspected concentration of 0.5–2.0 µg/mL in the CCK-8 assay. Pretreating with IEs, the viabilities of H_2_O_2_-insulted HepG2 cells were enhanced in a dose-dependent manner. All the IEs increased the cell viabilities in a sequence of root > flower > stem > leaf. The IEs at all assayed concentrations, especially at 2.0 μg/mL, were effective in reducing ROS and TBARS levels, restoring T-AOC, CAT, and GSH-PX activities in the cells that were affected due to H_2_O_2_, and showed with gradient changes as the concentration changed (Figure 4). The root and flower IEs exhibited relatively lower ROS and TBARS levels, whereas significantly higher CAT and GSH-PX activities than those of stem and leaf (*p* < 0.05). Taken together, the root and flower of *I. nervosa* Wall. possess distinguished antioxidant capacity from the stems and leaves.

Some early investigators hold the idea of a possible homeostatic equilibrium, in which the nutritional antioxidants would cause a decrease in endogenous antioxidant protection through feedback inhibition of regulatory mechanisms by increasing exogenous antioxidant capacity [26]. Instead, investigations on nutritional antioxidants, such as phenolic compounds, isothiocyanates, and other phytochemicals, actually observed an increased endogenous antioxidant protection, which was achieved by activating signal transduction pathways leading to altered gene expression, particularly in the Keap1/Nrf2/ARE pathway [26]. It is noteworthy that the root and flower IEs exhibited both the DPPH scavenging activities and increased intracellular antioxidant protection, indicating that the IEs would activate intracellular antioxidant signal pathways.

### 3.5. Effects of IEs on Nrf2/ARE Pathway Activation

Nrf2 is the main redox-sensitive transcription factor upregulating the expression of cell-protective genes in the oxidative stress response [28]. Once the Nrf2 signaling pathway is activated, Nrf2 is dissociated from Keap1-Nrf2 complex in the cytoplasm and transferred to the nucleus, where it binds to AREs in the promoter regions. Then, a series of detoxifying and antioxidant defense genes, such as GSTs, HO-1, NQO1, and MnSOD, are subsequently expressed to play anti-oxidative, anti-inflammatory, and anti-apoptotic roles [29]. GSH is a universal non-protein intracellular thiol present in millimolar concentrations, participating in the antioxidant process either directly by detoxifying ROS or indirectly via GSH-PX catalyzed reactions [30]. Glutamylcysteine synthase (GCL), which comprises of the catalytic subunit (GCLC) and the modulating subunit, is the rate-limiting enzyme in the GSH synthesis pathway [31].

**Figure 5:**
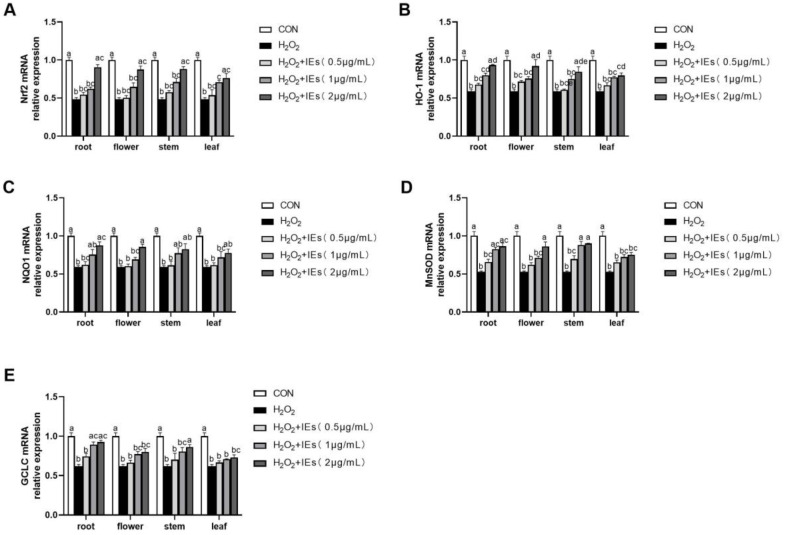
Effects of IEs on the mRNA expression of (**A**) Nrf2, (**B**) HO-1, (**C**) NQO1, (**D**) MnSOD, and (**E**) GCLC in H_2_O_2_-induced HepG2 cells. Values were presented as mean ± S.E.M. (*n* = 6). Bars within a figure without a common superscript differ significantly at *p* < 0.05.

In this context, the effects of IEs on the Nrf2/ARE pathway were examined via RT-PCR of Nrf2, HO-1, NQO1, MnSOD, and GCLC mRNA expression (Figure 5). All of the IEs relieved the impairment induced by H_2_O_2_, improving the Nrf2 and downstream genes’ HO-1, NQO1, MnSOD, and GCLC mRNA expression in a dose-dependent manner. The Nrf2 activation effect is relatively stronger in the root IE than in flower, stem, and leaf IEs at the concentration of 2.0 µg/mL. These results emphasized that the IEs can activate the Nrf2/ARE pathway to act as indirect antioxidants.

### 3.6. Correlations between Compound **1** and Antioxidant Parameters

**Figure 6:**
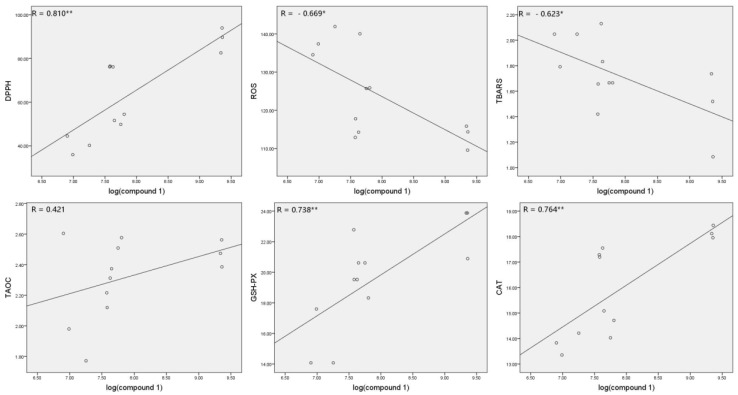
Correlations between the content of compound **1** (ion intensity at logarithmic scale) and antioxidant parameters (* *p* < 0.05; ** *p* < 0.01).

The chemical difference among IEs is responsible for their antioxidative activity variety. Compound **1** was illustrated as the most abundant and significant feature from the root extract. Herein, the correlations between compound **1** and antioxidant parameters were analyzed to reveal the relationships. As shown in Figure 6, the content of **1** has significantly positive correlations with DPPH, GSH-PX, and CAT activities (*p* < 0.01) and significantly negative correlation with ROS and TBARS levels (*p* < 0.05).

Compound **1** is a thymol *di*-isobutyrate without active phenolic hydroxyl group or proton to diminish DPPH radicals through a hydrogen atom transfer mechanism [32], a single electron transfer mechanism [33], or a sequential proton loss electron transfer mechanism [34,35]. Hence, the DPPH scavenging activity of compound **1** deserves further confirmation after its purification.

As mentioned earlier, the epoxy moiety of **1** could carry out a ring-opening and alkylation of one or more cysteine sulfhydryl groups of Keap1, leading to Nrf2 nuclear accumulation and upregulation of the expression of a large array of cytoprotective enzymes in response to oxidative assault. Human Keap1 contains 27 cysteines, some of which are purported to be the targets of electrophiles [36,37]. Cys151 has been reported as the most reactive site towards electrophilic natural products xanthohumol, isoliquiritigenin, and 10-shogaol [38]. The positive correlations between the content of **1** and GSH-PX and CAT activities indicate that compound **1** has a potential intracellular antioxidative effect which might be achieved by Nrf2 activation.

## 4. Conclusions

In summary, this study demonstrated that the application of comprehensive cheminformatics tools could facilitate the heterogeneity revelation in food chemistry and biological activities. By comparing the chemical space and antioxidative activities of ethanol extracts from root, stem, leaf, and flower parts of *I. nervosa* Wall., 10-isobutyryloxy-8,9-epoxythymol isobutyrate (**1**) was revealed as the most dominant and responsible marker from the root parts, that is, drawn from GNPS molecular networking, MolNetEnhancer, XCMS analysis, and `ili mapping. The direct and indirect antioxidative effects of IEs were found to correlate with the content of **1**. Moreover, the IEs were deduced to activate the Nrf2/ARE pathway to serve as indirect antioxidants. These findings also support the traditional application of the roots of *I. nervosa* Wall. as *Xiaoheiyao* and indicate **1** as one of the major functional factors, and it could be seen as a quality control factor as well. The results also cue the potential antioxidant of other parts, especially the flowers, and its toxicities need to be investigated before its application could be further studied.

## Figures and Tables

**Table 1:** Primers for Real-Time Reverse Transcription Polymerase Chain Reaction (RT-PCR).

Gene	Primer (5′→3′)	
	Forward	Reverse
β-actin	GTTGTCGACGACGAGCG	GCACAGAGCCTCGCCTT
Nrf2	TCCAGTCAGAAACCAGTGGAT	GAATGTCTGCGCCAAAAGCTG
NQO1	GTGGTGGAGTCGGACCTCTATG	AAGCCAGAACAGACTCGGCAG
HO-1	GAGTGTAAGGACCCATCGGA	GCCAGCAACAAAGTGCAAG
MnSOD	CCAGAAAATGCTATGATTGATATGAC	AAGGGAGATGTTACAGCCCAGATA
GCLC	AGGGAGTTTATCGCAAACCA	AAGTAACTCTGGGCATTCACA

**Table 2:** The hits in precursor tolerance against the GNPS library.

Compound	Cluster Index	Adduct	*m*/*z*	Mass Differ	Cosine Score	Shared Peaks	Identification
**1**	262	[M + H]^+^	320.997	0.173	0.84	6	10-Isobutyryloxy-8,9-epoxythymol isobutyrate
**2**	357	[M + H]^+^	354.996	0.004	0.97	7	Chlorogenic acid
**3**	374	[M-H_2_O + H]^+^	357.101	0.068	0.78	6	(2*S*,3*R*,3a*S*,7a*R*)-5-Allyl-2-(4-hydroxy-3-methoxyphenyl)-3a,7a-dimethoxy-3-methyl-3,3a,7,7a-tetrahydro-1-βenzofuran-6(2*H*)-one
**4**	414	[M + Na]^+^	365.172	0.068	0.86	7	Galactinol
**5**	1409	[M + Na]^+^	527.193	0.035	0.93	11	Melezitose
**6**	999	[M + Na]^+^	467.250	−1.954	0.81	6	(*Z*)-2,6-dimethyl-7-(4-methyl-5-oxooxolan-2-yl)-3-[[3,4,5-trihydroxy-6-(hydroxymethyl)oxan-2-yl]oxymethyl]hept-5-enoic acid
**7**	3462	[M + Na]^+^	691.309	2.099	0.82	8	Stachyose
**8**	6241	[M + Na]^+^	851.262	0.007	0.8	17	Polysaccharide Hexose x5
**9**	3590	[M + H]^+^	701.086	1.521	0.87	6	*N*-[(2*S*,3*R*,4*E*)-1-(β-d-Galactopyranosyloxy)-3-hydroxy-4-octadecen-2-yl]hexadecanamide
**10**	2202	[M + H]^+^	595.011	0.011	0.97	6	Kaempferol-7-*O*-neohesperidoside
**11**	824	[M + Na]^+^	432.993	−2.170	0.89	6	4-[(2*R*,3*R*,4*S*,5*S*,6*R*)-3,4,5-trihydroxy-6-(hydroxymethyl)oxan-2-yl]oxypentan-2-yl (*E*)-3-(4-hydroxyphenyl) prop-2-enoate
**12**	1112	[M + K]^+^	488.030	0.836	0.87	8	(2*R*,3*S*,4*S*,5*R*,6*R*)-2-[[(2*R*,3*R*,4*R*,5*S*)-3,4-dihydroxy-5-(hydroxymethyl)oxolan-2-yl]oxymethyl]-6-[(2*E*)-3,7-dimethylocta-2,6-dienoxy]oxane-3,4,5-triol
**13**	826	[M + H]^+^	435.064	1.954	0.78	10	Isovitexin
**14**	556	[M + H]^+^	384.929	0.785	0.9	7	Hydrastine
**15**	6464	[M + H]^+^	871.733	0.143	0.91	9	Pheophytin
**16**	2213	[M + H]^+^	595.418	2.149	0.81	6	Pheophorbide A

## Supplementary Materials

The supporting information is available on the online version. Table S1. The unique features from the root IE, Table S2. The unique features from the stem IE, Table S3. The unique features from the leaf IE, Table S4. The unique features from the flower IE, Table S5. The analog hits against GNPS library, Table S6. List of differential secondary metabolites from root, stem, leaf, and flower IEs, Table S7. List of differential secondary metabolites from root and stem IEs, Table S8. List of differential secondary metabolites from root and leaf IEs, Table S9. List of differential secondary metabolites from root and flower IEs.

## References

[1] Zhao L., Xin L.Q., Li Y.Q. (2007). Research advance of *Inula nervosa* Wall. in China. Food Drug.

[2] Yan L., Cheng X.R., Zeng Q., Qin J.J., Zhang W.D., Jin H.Z. (2001). Phytane and neoclerodane diterpenes from the aerial parts of *Inula nervosa* Wall. Biochem. Syst. Ecol..

[3] Yan L., Huang Y., Fu J.J., Qin J.J., Zeng Q., Zhu Y., Yan S.K., Zhang W.D., Jin H.Z. (2010). Three new phenylpropanoids from *Inula nervosa* Wall. Helv. Chim. Acta.

[4] Fujita H., Motokawa T., Katagiri T., Yokota S., Yamamoto A., Himeno M., Tanaka Y. (2009). Inulavosin, a melanogenesis inhibitor, leads to mistargeting of tyrosinase to lysosomes and accelerates its degradation. J. Invest. Dermatol..

[5] Cheng X.R., Zeng Q., Ren J., Qin J.J., Zhang S.D., Shen Y.H., Zhu J.X., Zhang F., Chang R.J., Zhu Y. (2011). Sesquiterpene lactones from *Inula falconeri*, a plant endemic to the Himalayas, as potential anti-inflammatory agents. Eur. J. Med. Chem..

[6] Cheng X.R., Zhang S.D., Wang C.H., Ren J., Qin J.J., Tang X., Shen Y.H., Yan S.K., Jin H.Z., Zhang W.D. (2013). Bioactive eudesmane and germacrane derivatives from *Inula wissmanniana* Hand.-Mazz. Phytochemistry.

[7] Wang G.W., Qin J.J., Cheng X.R., Shen Y.H., Shan L., Jin H.Z., Zhang W.D. (2014). *Inula* sesquiterpenoids: Structural diversity, cytotoxicity and anti-tumor activity. Expert Opin. Inv. Drug.

[8] Ministry of Health of PR China (2010). Announcement of the Ministry of Health of PR China No.9 of 2010. Chin. J. Food Hyg..

[9] Dinkova-Kostova A.T., Talalay P. (2008). Direct and indirect antioxidant properties of inducers of cytoprotective proteins. Mol. Nutr. Food Res..

[10] Ahn Y.H., Liu H., Wang X.J., Zhang Y., Stephenson K.K., Boronina T.N., Cole R.N., Dinkova-Kostova A.T., Talalay P., Cole P.A. (2010). Electrophilic tuning of the chemoprotective natural product sulforaphane. Proc. Natl. Acad. Sci. USA.

[11] Gersch M., Kreuzer J., Sieber S.A. (2012). Electrophilic natural products and their biological targets. Nat. Prod. Rep..

[12] Wang M., Carver J.J., Phelan V.V., Sanchez L.M., Garg N., Peng Y., Nguyen D.D., Watrous J., Kapono C.A., Luzzatto-Knaan T. (2016). Sharing and community curation of mass spectrometry data with Global Natural Products Social Molecular Networking. Nat. Biotechnol..

[13] Ernst M., Kang K.B., Caraballo-Rodriguez A.M., Nothias L.F., Wandy J., Chen C., Wang M., Rogers S., Medema M.H., Dorrestein P.C. (2019). MolNetEnhancer: Enhanced molecular networks by integrating metabolome mining and annotation tools. Metabolites.

[14] Shannon P., Markiel A., Ozier O., Baliga N.S., Wang J.T., Ramage D., Amin N., Schwikowski B., Ideker T. (2003). Cytoscape: A software environment for integrated models of biomolecular interaction networks. Genome Res..

[15] Gowda H., Ivanisevic J., Johnson C.H., Kurczy M.E., Benton H.P., Rinehart D., Nguyen T., Ray J., Kuehl J., Arevalo B. (2014). Interactive XCMS Online: Simplifying advanced metabolomic data processing and subsequent statistical analyses. Anal. Chem..

[16] Protsyuk I., Melnik A.V., Nothias L.F., Rappez L., Phapale P., Aksenov A.A., Bouslimani A., Ryazanov S., Dorrestein P.C., Alexandrov T. (2017). 3D molecular cartography using LC-MS facilitated by Optimus and `ili software. Nat. Protoc..

[17] Rosero J.C., Cruz S., Osorio C., Hurtado N. (2019). Analysis of phenolic composition of byproducts (seeds and peels) of Avocado (*Persea americana* Mill.) cultivated in Colombia. Molecules.

[18] Salla S., Sunkara R., Ogutu S., Walker L.T., Verghese M. (2016). Antioxidant activity of papaya seed extracts against H_2_O_2_ induced oxidative stress in HepG2 cells. LWT-Food Sci. Technol..

[19] Sohn S.H., Kim S.K., Kim Y.O., Kim H.D., Shin Y.S., Yang S.O., Kim S.Y., Lee S.W. (2013). A comparison of antioxidant activity of Korean White and Red Ginsengs on H2O2-induced oxidative stress in HepG2 hepatoma cells. J. Ginseng Res..

[20] https://gnps.ucsd.edu/ProteoSAFe/status.jsp?task=74fb92bf0553404ab0ffdc5376cfc44c.

[21] https://gnps.ucsd.edu/ProteoSAFe/status.jsp?task=a26a132027a242fc993df75766ec784d.

[22] Feunang Y.D., Eisner R., Knox C., Chepelev L., Hastings J., Owen G., Fahy E., Steinbeck C., Subramanian S., Bolton E. (2016). ClassyFire: Automated chemical classification with a comprehensive, computable taxonomy. J. Cheminform..

[23] Mohimani H., Gurevich A., Shlemov A., Mikheenko A., Korobeynikov A., Cao L., Shcherbin E., Nothias L.F., Dorrestein P.C., Pevzner P.A. (2018). Dereplication of microbial metabolites through database search of mass spectra. Nat. Commun..

[24] He A.N., She C.W., Zeng J.Y., Peng S.X. (2016). Comparison study on in vitro and in vivo antioxidant activities of *Inula nervosa* Wall. extracts from different parts. Chin. Pharmacol. Bull..

[25] Li K., Shi L.Z., Chen D., Hu Y.B., Lu M.F., Li R.C., Li S.X. (2013). Content determination of thymol and thymyl isobutyrate in *Inula nervosa* Wall from different parts, different habitats and different harvest periods by HPLC. J. Hunan Univ. Chin. Med..

[26] Forman H.J., Davies K.J., Ursini F. (2014). How do nutritional antioxidants really work: Nucleophilic tone and para-hormesis versus free radical scavenging in vivo. Free Radic. Biol. Med..

[27] Joko S., Watanabe M., Fuda H., Takeda S., Furukawa T., Hui S.-P., Shrestha R., Chiba H. (2017). Comparison of chemical structures and cytoprotection abilities between direct and indirect antioxidants. J. Funct. Foods.

[28] Jaiswal A.K. (2004). Nrf2 signaling in coordinated activation of antioxidant gene expression. Free Radicals Biol. Med..

[29] Lee C. (2017). Collaborative power of Nrf2 and PPARγ activators against metabolic and drug-induced oxidative injury. Oxid. Med. Cell. Longev..

[30] Teskey G., Abrahem R., Cao R., Gyurjian K., Islamoglu H., Lucero M., Martinez A., Paredes E., Salaiz O., Robinson B. (2018). Chapter five-Glutathione as a marker for human disease. Adv. Clin. Chem..

[31] Gipp J.J., Chang C., Mulcahy R.T. (1992). Cloning and nucleotide sequence of a full-length cDNA for human liver gamma-glutamylcysteine synthetase. Biochem. Biophys. Res. Commun..

[32] Litwinienko G., Ingold K.U. (2007). Solvent effects on the rates and mechanisms of reaction of phenols with free radicals. Acc. Chem. Rev..

[33] Galian R.E., Litwinienko G., Perez-Prieto J., Ingold K.U. (2007). Kinetic solvent effects on the reaction of an aromatic ketone π,π* triplet with phenol. Rate-retarding and rate-accelerating effects of hydrogen-bond acceptor solvents. J. Am. Chem. Soc..

[34] Litwinienko G., Ingold K.U. (2004). Abnormal solvent effects on hydrogen atom abstraction. 2. Resolution of the curcumin antioxidant controversy. The role of sequential proton loss electron transfer. J. Org. Chem..

[35] Litwinienko G., Ingold K.U. (2005). Abnormal solvent effects on hydrogen atom abstraction. 3. Novel kinetics in sequential proton loss electron transfer chemistry. J. Org. Chem..

[36] Eggler A.L., Liu G., Pezzuto J.M., van Breemen R.B., Mesecar A.D. (2005). Modifying specific cysteines of the electrophile-sensing human Keap1 protein is insufficient to disrupt binding to the Nrf2 domain Neh2. Proc. Natl. Acad. Sci. USA.

[37] Holland R., Hawkins A.E., Eggler A.L., Mesecar A.D., Fabris D., Fishbein J.C. (2008). Prospective type 1 and type 2 disulfides of Keap1 protein. Chem. Res. Toxicol..

[38] Luo Y., Eggler A.L., Liu D., Liu G., Mesecar A.D., van Breemen R.B. (2007). Sites of alkylation of human Keap1 by natural chemoprevention agents. J. Am. Soc. Mass Spectrom..

