# Supplementary material for: Chemical Space Charting of Different Parts of Inula nervosa Wall.: Upregulation of Expression of Nrf2 and Correlated Antioxidants Enzymes
Source: Molecules. 2020 Oct 19;25(20):4789. doi: 10.3390/molecules25204789 (PMC7587606; doi:10.3390/molecules25204789)
Supplement: Supplementary file 1 [file molecules-25-04789-s001.pdf]

Supporting information

Chemical Space Charting of Different Parts of *Inula nervosa* Wall.: upregulation of expression of Nrf2 and correlated antioxidants enzymes

Xiang-Rong Cheng[1,2,3*], Wei Zhao[1], Wen-Le Dong[1], Guo-Wei Le[1,2,3]

[1] School of Food Science and Technology, Jiangnan University, Wuxi, Jiangsu 214122, P.R. China

[2] National Engineering Research Center for Functional Food, Jiangnan University, Wuxi, Jiangsu 214122, P.R. China

[3] Collaborative Innovation Center of Food Safety and Quality Control in Jiangsu Province, Jiangnan University, Wuxi, Jiangsu 214122, P.R. China

**Table of Contents**	**Page**
Table S1. The unique features from the root IE	2
Table S2. The unique features from the stem IE	6
Table S3. The unique features from the leaf IE	8
Table S4. The unique features from the flower IE	10
Table S5. The analog hits against GNPS library	12
Table S6. List of differential secondary metabolites from root, stem, leaf, and flower IEs	14
Table S7. List of differential secondary metabolites from root and stem IEs	15
Table S8. List of differential secondary metabolites from root and leaf IEs	17
Table S9. List of differential secondary metabolites from root and flower IEs	19

Table S1. The unique features from the root IE

No.	Cluster index	*m/z*	Identification
1	3	145.134	unknown
2	43	225.051	Lactucin derivatives
3	51	228.08	unknown
4	53	233.063	9(10)-EpOME derivatives
5	55	233.075	[1,3]Benzodioxolo[5,6-e][2]benzazecin-14(6H)-one, 5,7,8,15-tetrahydro-3,4-dimethoxy-6-methyl- derivatives
6	57	235.026	1-O-trans-cinnamoyl-beta-D-glucopyranose derivatives
7	61	235.102	2-[4-(2-methylpropyl)phenyl]propanoic acid derivatives
8	62	235.123	Emodin derivatives
9	68	243.107	(+)-Chlorpheniramine derivatives
10	70	243.122	unknown
11	80	253.044	unknown
12	94	266.007	unknown
13	115	272.993	unknown
14	223	304.988	[2-[2-(2-methylpropanoyloxy)propan-2-yl]-7-oxo-2,3-dihydrofuro[3,2-g]chromen-3-yl] 2-methylpropanoate derivatives
15	226	306.933	unknown
16	227	307.047	3-(beta-D-glucopyranosyloxy)-2-methyl-4H-Pyran-4-one derivatives
17	259	318.965	Picrotin derivatives
18	276	322.988	fraxin derivatives
19	306	337.005	Sappanone A Dimethyl Ether derivatives
20	309	338.249	unknown
21	320	343.087	unknown
22	340	349.026	[2-[4-methyl-2-(2-methylpropanoyloxy)phenyl]oxiran-2-yl]methyl 2-methylpropanoate homologue
23	374	357.101	(2S,3R,3aS,7aR)-5-Allyl-2-(4-hydroxy-3-methoxyphenyl)-3a,7a-dimethoxy-3-methyl-3,3a,7,7a-tetrahydro-1-benzofuran-6(2H)-one
24	402	361.097	unknown
25	431	367.115	Rutarin homologue
26	432	367.111	unknown
27	434	367.112	Galactinol derivatives
28	436	367.112	[3,4,5-trihydroxy-6-[3,4,5-trihydroxy-6-(hydroxymethyl)oxan-2-yl]oxyoxan-2-yl]methyl (E)-2-methylbut-2-enoate derivatives
29	439	367.182	3-(beta-D-glucopyranosyloxy)-2-methyl-4H-Pyran-4-one derivatives
30	441	367.198	gerberinside derivatives
31	444	367.219	unknown
32	446	367.239	unknown
33	452	369.135	Melibiose derivatives

34	486	379.095	unknown
35	526	383.187	[(2S,3R,4S,5S,6R)-3,4,5-trihydroxy-6-(hydroxymethyl)oxan-2-yl] (2E,6E)-8-hydroxy-2,6-dimethylocta-2,6-dienoate derivatives
36	557	384.245	unknown
37	591	395.246	unknown
38	676	408.113	unknown
39	695	410.182	unknown
40	722	413.203	unknown
41	742	415.235	2-[6-Acetyl-3-(isobutyryloxy)-2,3-dihydro-1-benzofuran-2-yl]-2-propen-1-yl 2-methylpropanoate derivatives
42	796	427.282	unknown
43	837	437.132	unknown
44	924	453.208	Melezitose derivatives
45	925	453.222	Leupeptin derivatives
46	958	457.241	Cholic acid derivatives
47	966	460.046	unknown
48	976	464.902	unknown
49	981	465.176	(6aR,12aR)-6a,12a-Dihydro-6H-[1,3]dioxolo[5,6][1]benzofuro[3,2-c]chromen-3-yl-6-O-(carboxyacetyl)-beta-D-glucopyranoside derivatives
50	999	467.25	(Z)-2,6-dimethyl-7-(4-methyl-5-oxooxolan-2-yl)-3-[[3,4,5-trihydroxy-6-(hydroxymethyl)oxan-2-yl]oxymethyl]hept-5-enoic acid
51	1013	469.116	(2S)-2-[(2R,2'R,6'R,8a'S)-4,6'-Dihydroxy-2',5',5',8a'-tetramethyl-6-oxo-3',4',4a',5',6,6',7',8,8',8a'-decahydro-2'H-spiro[furo[2,3-e]isoindole-2,1'-naphthalen]-7(3H)-yl]pentanedioic acid derivatives
52	1123	488.136	unknown
53	1146	493.204	2-Phenylethyl 3-O-(4-carboxy-3-hydroxy-3-methylbutanoyl)-beta-D-glucopyranoside derivatives
54	1190	498.846	unknown
55	1246	507.051	Quercetin 3-O-malonylglucoside derivatives
56	1290	509.078	unknown
57	1299	511.225	Daidzin derivatives
58	1322	515.242	5-Hydroxy-1,3,3-trimethyl-2-(3-oxo-1-buten-1-yliden)cyclohexyl-6-O-[(2S,3R,4R)-3,4-dihydroxy-4-(hydroxymethyl)tetrahydro-2-furanyl]-β-D-glucopyranosid derivatives
59	1323	515.15	unknown
60	1371	520.395	2-Octadecanoyl-sn-glycero-3-phosphocholine derivatives
61	1379	522.539	unknown
62	1386	523.123	2-Methyl-4-oxo-4H-pyran-3-yl6-O-(4-carboxy-3-hydroxy-3-methylbutanoyl)-beta-D-glucopyranoside derivatives
63	1421	529.095	unknown
64	1423	529.216	Melezitose derivatives
65	1491	538.639	[(E,2R)-5-(3-chloro-5-formyl-2,6-dihydroxy-4-methylphenyl)-3-

			methyl-1-[(1S,2R,6R)-1,2,6-trimethyl-3-oxocyclohexyl]pent-3-en-2-yl] 3-methylbutanoate derivatives
66	1506	539.07	unknown
67	1574	545.227	(Z)-2,6-dimethyl-7-(4-methyl-5-oxooxolan-2-yl)-3-[[3,4,5-trihydroxy-6-(hydroxymethyl)oxan-2-yl]oxymethyl]hept-5-enoic acid derivatives
68	1799	560.163	Glucopiericidin C derivatives
69	1826	561.122	(Z)-2,6-dimethyl-7-(4-methyl-5-oxooxolan-2-yl)-3-[[3,4,5-trihydroxy-6-(hydroxymethyl)oxan-2-yl]oxymethyl]hept-5-enoic acid derivatives
70	1964	571.291	unknown
71	1982	571.355	unknown
72	2016	575.203	(3S,4R,6aR,6bS,8aS,11R,12R,14bR)-3,12-dihydroxy-4,6a,6b,11,12,14b-hexamethyl-8a-[3,4,5-trihydroxy-6-(hydroxymethyl)oxan-2-yl]oxycarbonyl-1,2,3,4a,5,6,7,8,9,10,11,12a,14,14a-tetradecahydropicene-4- carboxylic acid derivatives
73	2051	579.201	unknown
74	2147	591.143	Cascaroside B derivatives
75	2206	595.177	unknown
76	2230	599.157	Lysophosphatidylserine derivatives
77	2338	609.159	unknown
78	2393	613.237	unknown
79	2407	615.254	Stachyose derivatives
80	2410	615.44	unknown
81	2442	617.433	unknown
82	2574	629.436	unknown
83	2578	629.138	unknown
84	2587	629.459	unknown
85	2600	631.272	unknown
86	2604	631.42	unknown
87	2608	631.531	unknown
88	2623	634.244	Cascaroside B derivatives
89	2669	637.308	unknown
90	2678	637.426	unknown
91	2738	643.332	unknown
92	2769	647.174	unknown
93	2843	651.471	unknown
94	3158	671.302	unknown
95	3162	671.385	unknown
96	3422	689.199	3-[[6-[[1,4a-dimethyl-6-methylidene-5-[2-(5-oxo-2H-furan-4-yl)ethyl]-3,4,5,7,8,8a-hexahydro-2H-naphthalen-1-yl]methoxy]-3,4,5-trihydroxyoxan-2-yl]methoxy]-3-oxopropanoic acid derivatives
97	3462	691.309	Stachyose

98	3668	703.169	unknown
99	3692	705.164	unknown
100	4264	733.044	Stachyose derivatives
101	4591	751.219	euphodendroidin B homologue
102	4628	753.105	unknown
103	4911	769.14	unknown
104	5019	773.131	unknown
105	5061	775.118	unknown
106	5252	787.2	unknown
107	5654	813.74	unknown
108	5827	823.267	unknown
109	6022	837.133	unknown
110	6205	849.202	unknown
111	6230	851.209	Polysaccharide Hexose x5
112	6241	851.262	Polysaccharide Hexose x5
113	6701	895.257	1-stearoyl-2-(8Z,11Z,14Z-icosatrienoyl)-sn-glycero-3-phosphoserine derivatives
114	7398	968.958	unknown
115	7497	1013.23	unknown
116	7503	1054.97	unknown
117	7547	1175.23	unknown
118	7559	1485.85	unknown

Table S2. The unique features from the stem IE

No.	Cluster index	*m/z*	Identification
1	125	277.217	Abrine derivatives
2	147	291.095	unknown
3	152	293.066	unknown
4	347	353.01	1-Arachidonoylglycerol derivatives
5	417	365.136	1-[2,6-dihydroxy-4-[(2S,3R,4S,5S,6R)-3,4,5-trihydroxy-6-(hydroxymethyl)oxan-2-yl]oxyphenyl]ethanone derivatives
6	468	373.203	unknown
7	956	457.255	α-D-Glucopyranoside,4-O-[(2E)-1-oxo-3-phenyl-2-propen-1-yl]-beta-D-fructofuranosyl derivatives
8	962	457.4	unknown
9	1010	469.039	unknown
10	1020	470.342	4-[2-(2,6-dimethoxy-4-prop-2-enylphenoxy)-1-hydroxypropyl]-2-methoxyphenol derivatives
11	1063	479.103	2-Phenylethyl 3-O-(4-carboxy-3-hydroxy-3-methylbutanoyl)-beta-D-glucopyranoside derivatives
12	1310	512.244	7-O-beta-glucopyranosyl-4'-hydroxy-5-methoxyisoflavone derivatives
13	1348	517.216	(1β,2α,9ξ,11β,12α,15β)-1,2,11,12,14,15-Hexahydroxypicras-4-en-16- one derivatives
14	1417	528.727	unknown
15	1461	533.041	unknown
16	1596	547.146	unknown
17	1651	553.329	unknown
18	1811	561.147	[(2R,3S,4S,5S)-4-hydroxy-2,5-bis(hydroxymethyl)-5-[(2R,3R,4S,5S,6R)-3,4,5-trihydroxy-6-(hydroxymethyl)oxan-2-yl]oxyoxolan-3-yl] (E)-3-phenylprop-2-enoate derivatives
19	1931	567.09	unknown
20	2213	595.418	Pheophorbide A
21	2314	607.221	unknown
22	2319	607.25	[(2R)-3-[2-aminoethoxy(hydroxy)phosphoryl]oxy-2-[(Z)-hexadec-9-enoyl]oxypropyl] (Z)-hexadec-9-enoate derivatives
23	2406	615.269	unknown
24	2441	617.396	unknown
25	2613	633.213	unknown
26	2625	635.014	unknown
27	3421	689.258	unknown
28	3565	699.167	unknown
29	3698	705.131	unknown
30	3703	705.301	unknown
31	4190	727.313	unknown
32	4452	742.834	unknown

33	4815	762.232	unknown
34	4880	767.257	unknown
35	4928	769.266	unknown
36	4985	771.28	unknown
37	4990	771.313	unknown
38	5004	771.388	unknown
39	5068	775.182	unknown
40	5182	783.254	unknown
41	5216	785.207	unknown
42	5266	787.538	unknown
43	5423	799.158	unknown
44	6555	879.795	unknown
45	7128	938.116	unknown
46	7490	995.214	unknown
47	7491	995.261	unknown
48	7538	1131.74	unknown
49	7542	1153.7	unknown
50	7543	1159.66	unknown
51	7549	1181.82	unknown

Table S3. The unique features from the leaf IE

No.	Cluster index	*m/z*	Identification
1	15	181.048	unknown
2	20	197.05	Lepargylic acid derivatives
3	85	257.06	unknown
4	300	334.321	unknown
5	325	345.114	unknown
6	327	347.032	unknown
7	412	363.082	2,8-Dihydroxy-5,5,8-trimethyl-11-oxatetracyclo [7.3.1.0~1,9~.0~3,7~]tridecan-10-one derivatives
8	556	384.929	Hydrastine
9	1108	486.124	unknown
10	1917	566.421	unknown
11	2379	611.407	unknown
12	2432	617.196	unknown
13	2529	625.385	unknown
14	2687	639.3	[(2R)-2-hexadecanoyloxy-3-tetradecanoyloxypropyl] 2-(trimethylazaniumyl)ethyl phosphate derivatives
15	2710	641.354	unknown
16	3013	661.421	unknown
17	3029	662.409	[(2R)-3-[2-aminoethoxy(hydroxy)phosphoryl]oxy-2-[(Z)-hexadec-9-enoyl]oxypropyl] hexadecanoate derivatives
18	3209	675.374	unknown
19	3211	675.465	unknown
20	3330	683.4	16-Deacetylgeyerline derivatives
21	3539	697.351	Methyllycaconitine homologue
22	4203	727.538	unknown
23	4328	737.117	unknown
24	4333	737.446	unknown
25	4447	742.475	[(2R)-1-[2-aminoethoxy(hydroxy)phosphoryl]oxy-3-[(Z)-octadec-1-enoxy]propan-2-yl] (8Z,11Z,14Z)-icosa-8,11,14-trienoate derivatives
26	4694	757.476	unknown
27	4874	766.895	unknown
28	5542	806.429	N-[(2S,3R)-3-hydroxy-1-[(2R,5R,6R)-3,4,5-trihydroxy-6-(hydroxymethyl)oxan-2-yl]oxyoctadecan-2-yl]tetracosanamide derivatives
29	5716	815.579	[(2R)-2,3-di(dodecanoyloxy)propyl](9Z,12Z)-octadeca-9,12-dienoate derivatives
30	5806	821.367	unknown
31	5869	827.597	unknown
32	5903	829.412	unknown
33	6092	842.348	unknown
34	6456	871.323	(2S)-2-amino-3-[hydroxy-[(2R)-2-[(9Z,12Z)-octadeca-9,12-die

			noyl] oxy-3-[(Z)-octadec-9-enoyl]oxypropoxy]phosphoryl]oxypropanoic acid derivatives
35	6464	871.733	Pheophytin
36	6467	872.621	Pheophytin
37	6473	872.595	euphodendroidin K derivatives
38	6503	874.787	unknown
39	6590	883.444	unknown
40	6645	887.467	Chiococcasaponin IV derivatives
41	6720	899.143	unknown
42	6886	913.44	unknown
43	7131	937.707	Diglycosyl diacylglycerol C31 derivatives
44	7508	1091.55	unknown
45	7509	1091.65	unknown
46	7511	1091.73	unknown
47	7513	1092.56	unknown
48	7515	1101.54	unknown
49	7533	1107.72	unknown
50	7534	1107.76	unknown
51	7550	1205.59	unknown
52	7552	1224.82	unknown

Table S4. The unique features from the flower IE

No.	Cluster index	*m/z*	Identification
1	24	198.103	3,5-Dinitrosalicylate derivatives
2	65	236.059	unknown
3	362	355.151	unknown
4	521	383.251	Coprostanone homologue
5	623	399.224	unknown
6	780	425.238	unknown
7	973	463.259	unknown
8	980	465.147	unknown
9	1047	477.191	unknown
10	1059	478.995	Aloenin derivatives
11	1144	492.987	[(2R)-3-[2-aminoethoxy(hydroxy)phosphoryl]oxy-2-hydroxypropyl] (7Z,10Z,13Z,16Z)-docosa-7,10,13,16-tetraenoate derivatives
12	1145	493.088	[(2R)-3-[2-aminoethoxy(hydroxy)phosphoryl]oxy-2-hydroxypropyl] (Z)-octadec-9-enoate derivatives
13	1155	495.092	unknown
14	1156	495.181	unknown
15	1359	519.039	Ambocin derivatives
16	1364	519.287	β-D-Glucopyranoside,(1R)-1-ethenyl-1,5-dimethyl-4-hexen-1-yl 6-O-alpha-L-arabinopyranosyl- derivatives
17	1501	539.213	5,7-dihydroxy-2-[3-hydroxy-4-[(2S,3R,4S,5S,6R)-3,4,5-trihydroxy-6-(hydroxymethyl)oxan-2-yl]oxyphenyl]chromen-4-one derivatives
18	1535	541.078	(6aR,12aR)-6a,12a-Dihydro-6H-[1,3]dioxolo[5,6][1]benzofuro[3,2-c]chromen-3-yl6-O-(carboxyacetyl)-beta-D-glucopyranoside derivatives
19	1538	541.25	5,7-dihydroxy-2-[4-hydroxy-3-[(2S,3R,4S,5R)-3,4,5-trihydroxyoxan-2-yl]oxyphenyl]-3-methoxychromen-4-one derivatives
20	1613	549.093	3-[(2S,3R,4S,5S,6R)-4,5-dihydroxy-6-(hydroxymethyl)-3-[(2S,3R,4S,5S,6R)-3,4,5-trihydroxy-6-(hydroxymethyl)oxan-2-yl]oxyoxan-2-yl]oxy-2-(3,4-dihydroxyphenyl)-5-hydroxy-7-methoxychromen-4-one derivatives
21	1614	549.244	3-[(2S,3R,4S,5S,6R)-4,5-dihydroxy-6-(hydroxymethyl)-3-[(2S,3R,4S,5R)-3,4,5-trihydroxyoxan-2-yl]oxyoxan-2-yl]oxy-2-(3,4-dihydroxyphenyl)-5-hydroxy-7-methoxychromen-4-one derivatives
22	1775	557.119	unknown
23	1831	561.122	unknown
24	1845	561.241	Polysaccharide Hexose x3 derivatives
25	1889	563.117	Isorhamnetin 3-galactoside derivatives
26	2088	584.198	Vitexin-2"-O-rhamnoside derivatives
27	2330	608.972	Kaempferol-7-O-neohesperidoside homologue

28	2433	617.115	5-hydroxy-3-(5-hydroxy-2,4-dimethoxyphenyl)-6-methoxy-7-[3,4,5-trihydroxy-6-(hydroxymethyl)oxan-2-yl]oxychromen-4-one derivatives
29	2551	627.082	[(2S,3R,4S,5S,6R)-3,4,5-trihydroxy-6-(hydroxymethyl)oxan-2-yl] (4aS,6aS,6bR,8R,9R,10R,11R,12aR)-8,10,11-trihydroxy-9-(hydroxymethyl)-2,2,6a,6b,9,12a-hexamethyl-1,3,4,5,6,6a,7,8,8a,10,11,12,13,14b-tetradecahydropicene-4a-carboxylate derivatives
30	2955	656.995	Cascaroside B derivatives
31	3069	665.229	unknown
32	3328	683.189	unknown
33	3400	687.362	unknown
34	4585	750.727	unknown
35	5077	775.315	unknown
36	5503	803.205	unknown
37	5751	817.216	1-Arachidonoyl-2-docosahexaenoyl-sn-glycero-3-phosphoethanolamine derivatives
38	6162	845.834	unknown
39	6908	917.22	unknown
40	7110	937.868	unknown

Table S5. The analog hits against GNPS library

Compd.	Cluster Index	Adduct	*m/z*	Mass differ	Cosine score	Shared peaks	Identification
**17**	1059	$[M+Na]^+$	478.995	46	0.98	6	Aloenin derivatives
**18**	917	$[M+Na]^+$	449.028	16	0.98	6	Aloenin derivatives
**19**	2330	$[M+H]^+$	608.972	14	0.97	6	Kaempferol-7-O-neohesperidoside homologue
**20**	2049	$[M+H]^+$	579.045	-16	0.97	6	Kaempferol-7-O-neohesperidoside derivatives
**21**	2181	$[M+Na]^+$	593.357	31	0.97	6	2-Thio PAF derivatives
**22**	4047	$[M+Na]^+$	719.071	30	0.95	9	Stachyose derivatives
**23**	1574	$[M+Na]^+$	545.227	76	0.95	8	(Z)-2,6-dimethyl-7-(4-methyl-5-oxooxolan-2-yl)-3-[[3,4,5-trihydroxy-6-(hydroxymethyl)oxan-2-yl] oxymethyl]hept-5-enoic acid derivatives
**24**	585	$[M+H]^+$	394.296	-19	0.95	7	4-(β-D-Glucopyranosyloxy)-2-pentanyl (2E)-3-(4-hydroxyphenyl)acrylate derivatives
**25**	4264	$[M+Na]^+$	733.044	44	0.94	7	Stachyose derivatives
**26**	515	$[M+Na]^+$	383.161	14	0.94	6	[(2S,3R,4S,5S,6R)-3,4,5-trihydroxy-6-(hydroxymethyl)oxan-2-yl] (2E,6E)-8-hydroxy-2,6-dimethylocta-2,6-dienoate homologue
**27**	227	$[M+H]^+$	307.047	18	0.94	6	2-Methyl-4-oxo-4H-pyran-3-yl β-D-glucopyranoside derivatives
**28**	358	$[M+NH_4]^+$	354.939	-97	0.93	8	[(2R,3S,4S,5R,6S)-3,4,5-trihydroxy-6-(4-hydroxyphenoxy)oxan-2-yl]methyl (E)-3-(3,4-dihydroxyphenyl)prop-2-enoate derivatives
**29**	2407	$[M+Na]^+$	615.254	-74	0.93	8	Stachyose derivatives
**30**	1889	$[M+H]^+$	563.117	84	0.93	6	Isorhamnetin 3-galactoside derivatives
**31**		$[M+NH_4]^+$	354.939	14	0.93	7	[(2R,3S,4S,5R,6S)-3,4,5-trihydroxy-6-(4-hydroxyphenoxy)oxan-2-yl]methyl (E)-3-(3,4-dihydroxyphenyl)prop-2-enoate homologue
**32**	662	$[M+H]^+$	407.034	-40	0.93	7	(2S,3S,4S,5R,6S)-3,4,5-trihydroxy-6-[5-hydroxy-2-(4-hydroxyphenyl)-4-oxochromen-7-yl]oxyoxane-2-carboxylic acid derivatives
**33**	1538	$[M+H]^+$	541.25	92	0.93	7	5,7-dihydroxy-2-[4-hydroxy-3-[(2S,3R,4S,5R)-3,4,5-trihydroxyoxan-2-yl]oxyphenyl]-3-methoxychromen-4-one derivatives
**34**	970	$[M-H]^-$	463.038	74	0.93	6	(2S,3R,4S,5S,6R)-2-[3-hydroxy-5-[(Z)-2-(4-hydroxyphenyl)ethenyl]phenoxy]-6-(hydroxymethyl)oxane-3,4,5-triol derivatives
**35**	2186	$[M+FA-H]^-$	593.361	14	0.92	8	(1α,2α,3β,5ξ,9ξ,13ξ,14ξ,18ξ)-1,2,3,22,23,29-Hexahydroxy-13,27-cycloolean-11-en-28-oic acid homologue
**36**	1552	$[M+Na]^+$	543.136	16	0.91	6	Melezitose derivatives
**37**	4059	$[M+Na]^+$	719.095	30	0.9	9	Stachyose derivatives
**38**	1359	$[M+H]^+$	519.039	-46	0.9	7	Ambocin derivatives
**39**	1299	$[M+H]^+$	511.225	94	0.9	7	Daidzin derivatives
**40**	223	$[M+H]^+$	304.988	-98	0.89	6	[2-[2-(2-methylpropanoyloxy)propan-2-yl]-7-oxo-2,3-dihydrofuro[3,2-g]chromen-3-yl] 2-methylpropanoate homologue
**41**	1496	$[M+Na]^+$	539.101	70	0.89	6	(Z)-2,6-dimethyl-7-(4-methyl-5-oxooxolan-2-yl)-3-[[3,4,5-trihydroxy-6-(hydroxymethyl)oxan-2-yl]oxymethyl]hept-5-enoic acid derivatives
**42**	2955	$[M+Na]^+$	656.995	54	0.89	7	Cascaroside B derivatives
**43**	57	$[M-H]^-$	235.026	-74	0.89	7	1-O-trans-cinnamoyl-beta-D-glucopyranose derivatives

**44**	805	$[M+Na]^+$	429.205	86	0.88	8	2-{2-[(Isobutyryloxy)methyl]-2-oxiranyl}-5-methylphenyl 2-methylpropanoate derivatives
**45**	981	$[M+Na]^+$	465.176	-90	0.88	9	(6aR,12aR)-6a,12a-Dihydro-6H-[1,3]dioxolo[5,6][1]benzofuro[3,2-c]chromen-3-yl 6-O-(carboxyacetyl)-beta-D-glucopyranoside derivatives
**46**	1501	$[M+H]^+$	539.213	90	0.88	6	5,7-dihydroxy-2-[3-hydroxy-4-[(2S,3R,4S,5S,6R)-3,4,5-trihydroxy-6-(hydroxymethyl)oxan-2-yl]oxyphenyl]chromen-4-one derivatives
**47**	925	$[M+H]^+$	453.222	26	0.88	6	Leupeptin derivatives
**48**	971	$[M+H]^+$	463.031	44	0.88	6	3-[(2R,3S,4S,5S)-3,4-dihydroxy-5-(hydroxymethyl)oxolan-2-yl]oxy-5,7-dihydroxy-2-(4-hydroxyphenyl)chromen-4-one derivatives
**49**	441	$[M+Na]^+$	367.198	6	0.87	8	gerberinside derivatives
**50**	924	$[M+Na]^+$	453.208	-74	0.86	9	Melezitose derivatives
**51**	3539	$[M+H]^+$	697.351	14	0.86	8	Methyllycaconitine homologue
**52**	526	$[M-H]^-$	383.187	38	0.86	6	[(2S,3R,4S,5S,6R)-3,4,5-trihydroxy-6-(hydroxymethyl)oxan-2-yl] (2E,6E)-8-hydroxy-2,6-dimethylocta-2,6-dienoate derivatives
**53**	2147	$[M+Na]^+$	591.143	-12	0.86	6	Cascaroside B derivatives
**54**	477	$[M-H]^-$	377.091	24	0.85	6	Chlorogenic acid Hemihydrate derivatives
**55**	4058	$[M+Na]^+$	719.193	30	0.85	6	Stachyose derivatives
**56**	2016	$[M-H]^-$	575.203	88	0.85	9	(3S,4R,6aR,6bS,8aS,11R,12R,14bR)-3,12-dihydroxy-4,6a,6b,11,12,14b-hexamethyl-8a-[3,4,5-trihydroxy-6-(hydroxymethyl)oxan-2-yl]oxycarbonyl-1,2,3,4a,5,6,7,8,9,10,11,12a,14,14a-tetradecahydropicene-4-carboxylic acid derivatives
**57**	340	$[M+H]^+$	349.026	28	0.84	6	[2-[4-methyl-2-(2-methylpropanoyloxy)phenyl]oxiran-2-yl]methyl 2-methylpropanoate homologue
**58**	2623	$[M+Na]^+$	634.244	31	0.84	7	Cascaroside B derivatives
**59**	2088	$[M+H]^+$	584.198	5	0.84	7	Vitexin-2"-O-rhamnoside derivatives
**60**	1182	$[M+H]^+$	499.132	28	0.83	8	[2,6-dihydroxy-5-[3,4,5-trihydroxy-6-(hydroxymethyl)oxan-2-yl]oxycyclohex-3-en-1-yl] (E)-3-(3,4-dihydroxyphenyl)prop-2-enoate homologue
**61**	3420	$[M+Na]^+$	689.218	72	0.83	6	Saponarin derivatives
**62**	276	$[M-H]^-$	322.988	-46	0.82	6	fraxin derivatives
**63**	732	$[M-H]^-$	415.085	62	0.81	7	xanthohumol derivatives
**64**	1799	$[M+H]^+$	560.163	12	0.8	8	Glucopiericidin C derivatives
**65**	75	$[M+H]^+$	249.029	56	0.79	6	Quinic acid homologue
**66**	4591	$[M+Na]^+$	751.219	14	0.79	6	euphodendroidin B homologue
**67**	334	$[M+NH_4]^+$	346.352	50	0.79	6	3-(5,7-dimethoxy-4-oxochromen-2-yl)propanoic acid derivatives
**68**	431	$[M-H]^-$	367.115	-56	0.76	6	Rutarin homologue
**69**	5067	$[M+Na]^+$	775.278	5	0.76	8	Microcolin A derivatives
**70**	521	$[M-H_2O+H]^+$	383.251	14	0.72	10	Coprostanone homologue

Table S6. List of differential secondary metabolites from root, stem, leaf, and flower IEs

No.	ID	pvalue	qvalue	CV	m/z	identification
1	M1056T11	0.00051	0.00257	0.176	1055.91	
2	M163T11	0.00051	0.00257	0.174	163.11	
3	M540T11	0.00051	0.00257	0.278	540.08	
4	M116T2	0.00051	0.00257	0.346	116.12	
5	M539T11_2	0.00051	0.00257	0.427	539.10	
6	M132T2	0.00051	0.00257	0.435	132.06	
7	M355T9	0.00051	0.00257	0.475	355.16	**2**
8	M539T11_1	0.00051	0.00257	0.493	539.06	
9	M382T2	0.00058	0.00257	0.242	382.08	
10	M500T11	0.00062	0.00257	0.331	500.12	
11	M543T2	0.00076	0.00257	0.288	543.13	
12	M104T2	0.00076	0.00257	0.457	104.17	
13	M449T10_1	0.00076	0.00257	0.490	449.04	
14	M595T10	0.00091	0.00263	0.458	595.04	**10**
15	M1055T11	0.00118	0.00271	0.234	1054.85	
16	M487T9	0.00123	0.00272	0.253	487.23	**12**
17	M321T20_2	0.00127	0.00272	0.035	320.99	**1**
18	M365T2	0.00146	0.00276	0.183	365.19	**3**
19	M705T2	0.00146	0.00276	0.415	705.10	
20	M719T11	0.00188	0.00281	0.186	719.05	
21	M453T10_1	0.00212	0.00283	0.180	453.17	
22	M499T11_2	0.00212	0.00283	0.470	499.11	
23	M479T10	0.00234	0.00285	0.402	479.01	
24	M381T2_2	0.00255	0.00286	0.126	381.08	
25	M787T14	0.00255	0.00286	0.480	787.27	
26	M233T19	0.00274	0.00287	0.193	233.06	
27	M381T2_1	0.00275	0.00287	0.130	381.03	
28	M383T3	0.00292	0.00288	0.171	383.16	
29	M453T10_2	0.00292	0.00288	0.230	453.23	
30	M527T2_1	0.00310	0.00288	0.295	527.17	**5**
31	M322T20_2	0.00322	0.00289	0.185	322.03	
32	M527T2_2	0.00330	0.00289	0.455	527.22	
33	M520T11	0.00408	0.00346	0.349	520.13	
34	M145T20	0.00483	0.00401	0.085	145.20	

$p < 0.01$, $q < 0.01$, CV < 0.5

Table S7. List of differential secondary metabolites from root and stem IEs

No.	ID	Fold change	log2fold	Updown	*p* value	*q* value	*m/z*	Identification
1	M104T2	2.46	1.30	UP	0.001246	6.83E-05	104.17	
2	M355T9	5.27	2.40	UP	6.35E-08	1.81E-07	355.16	
3	M166T2	13.97	3.80	UP	5.01E-05	7.81E-06	166.05	
4	M132T2	15.27	3.93	UP	8.19E-05	9.35E-06	132.06	
5	M381T2_2	2.23	-1.16	DOWN	6.15E-05	8.39E-06	381.08	
6	M382T2	2.41	-1.27	DOWN	0.000968	5.55E-05	382.08	
7	M487T9	3.57	-1.84	DOWN	0.000339	2.38E-05	487.23	
8	M543T2	4.37	-2.13	DOWN	0.000844	4.96E-05	543.13	
9	M499T11_2	4.93	-2.30	DOWN	2.35E-05	5.6E-06	499.09	
10	M1055T11	5.07	-2.34	DOWN	7.76E-05	9.18E-06	1054.84	
11	M500T11	5.22	-2.38	DOWN	0.004172	0.000183	500.13	
12	M719T11	5.39	-2.43	DOWN	0.000382	2.6E-05	719.05	
13	M705T2	8.85	-3.15	DOWN	0.003184	0.000145	705.10	
14	M349T22	13.32	-3.74	DOWN	1.19E-05	4.05E-06	349.02	
15	M293T18	15.77	-3.98	DOWN	2.69E-05	5.91E-06	292.95	
16	M383T3	16.81	-4.07	DOWN	3.55E-05	6.78E-06	383.16	
17	M253T14	19.82	-4.31	DOWN	0.000334	2.36E-05	253.02	
18	M329T22	21.86	-4.45	DOWN	0.000315	2.27E-05	329.13	
19	M365T2	24.91	-4.64	DOWN	4.67E-05	7.6E-06	365.19	**4**
20	M344T20	25.87	-4.69	DOWN	0.000201	1.68E-05	344.17	
21	M415T22	26.72	-4.74	DOWN	0.007119	0.000301	415.24	
22	M323T20_2	28.39	-4.83	DOWN	0.000114	1.17E-05	323.02	
23	M163T20	29.11	-4.86	DOWN	0.000597	3.71E-05	163.08	
24	M219T21	32.66	-5.03	DOWN	1.05E-05	3.76E-06	219.03	
25	M453T10_1	34.84	-5.12	DOWN	4.3E-05	7.36E-06	453.17	
26	M337T21	34.86	-5.12	DOWN	0.00087	5.09E-05	337.03	
27	M233T19	35.88	-5.17	DOWN	5.97E-05	8.31E-06	233.06	
28	M277T20	35.91	-5.17	DOWN	3.81E-07	3.15E-07	277.23	
29	M321T20_2	40.06	-5.32	DOWN	4.52E-07	3.22E-07	320.99	**1**
30	M231T21	40.36	-5.33	DOWN	0.001355	7.3E-05	231.13	
31	M322T20_2	41.02	-5.36	DOWN	4.91E-05	7.75E-06	322.03	
32	M453T10_2	43.36	-5.44	DOWN	0.000141	1.34E-05	453.23	
33	M145T20	43.98	-5.46	DOWN	9.33E-07	5.33E-07	145.20	
34	M323T20_1	46.61	-5.54	DOWN	0.001739	9.02E-05	322.96	
35	M322T20_1	46.74	-5.55	DOWN	1.08E-05	3.83E-06	321.98	
36	M424T21	46.98	-5.55	DOWN	0.000502	3.19E-05	424.13	
37	M527T2_2	47.69	-5.58	DOWN	0.003259	0.000148	527.22	
38	M251T15	48.03	-5.59	DOWN	0.000233	1.85E-05	251.05	
39	M407T21	56.01	-5.81	DOWN	0.000104	1.1E-05	407.01	
40	M357T21	56.35	-5.82	DOWN	0.000313	2.26E-05	357.11	**3**
41	M527T2_1	56.58	-5.82	DOWN	0.000161	1.45E-05	527.17	**5**
42	M689T2	66.36	-6.05	DOWN	0.000166	1.48E-05	689.22	

43	M429T21	77.81	-6.28	DOWN	0.002804	0.000132	429.21
44	M305T21	79.18	-6.31	DOWN	0.008063	0.000337	304.98
45	M217T21	79.70	-6.32	DOWN	0.000448	2.93E-05	217.14
46	M289T20	85.49	-6.42	DOWN	0.002799	0.000132	289.23
47	M343T20_1	85.81	-6.42	DOWN	1.91E-05	5.14E-06	343.05
48	M115T20	86.63	-6.44	DOWN	0.000985	5.62E-05	115.22
49	M146T20	88.60	-6.47	DOWN	0.000346	2.41E-05	146.21
50	M135T20	91.61	-6.52	DOWN	0.001999	0.000101	135.15
51	M121T21	91.80	-6.52	DOWN	0.000213	1.75E-05	121.16
52	M343T20_2	99.47	-6.64	DOWN	0.005432	0.000235	343.13
53	M225T12	135.20	-7.08	DOWN	0.003154	0.000144	225.06
54	M307T22	149.93	-7.23	DOWN	0.000102	1.08E-05	306.92
55	M651T13	159.48	-7.32	DOWN	0.001849	9.49E-05	651.47
56	M149T22	160.71	-7.33	DOWN	2.25E-05	5.5E-06	149.08
57	M335T21	180.28	-7.49	DOWN	8.01E-05	9.28E-06	335.00
58	M145T21	327.42	-8.36	DOWN	3.74E-07	3.14E-07	145.21
59	M336T21	447.52	-8.81	DOWN	6.18E-05	8.41E-06	336.03
60	M616T12	471.90	-8.88	DOWN	0.000142	1.35E-05	616.43
61	M430T21	525.64	-9.04	DOWN	0.003431	0.000153	430.20
62	M219T22	574.18	-9.17	DOWN	0.001174	6.51E-05	218.98
63	M308T22	582.08	-9.19	DOWN	0.002316	0.000114	307.93
64	M630T13	1343.17	-10.39	DOWN	0.000232	1.84E-05	630.44
65	M629T13	1441.12	-10.49	DOWN	8.05E-05	9.3E-06	629.42
66	M615T12	1882.61	-10.88	DOWN	1.99E-05	5.23E-06	615.43

fold change > 2, $p < 0.01$, $q < 0.01$

Table S8. List of differential secondary metabolites from root and leaf IEs

No.	ID	Fold change	log2fold	Updown	*p* value	*q* value	*m/z*	Identification
1	M321T20_1	210.08	-7.71	DOWN	3.03E-09	3.52E-09	320.96	**1**
2	M145T21	9751.03	-13.25	DOWN	3.8E-07	2.21E-07	145.21	
3	M145T20	438.86	-8.78	DOWN	1.59E-06	6.15E-07	145.19	
4	M615T12	2271.88	-11.15	DOWN	8.32E-06	2.09E-06	615.44	
5	M381T2	687.46	-9.43	DOWN	1.08E-05	2.41E-06	381.09	
6	M322T20	367.11	-8.52	DOWN	1.99E-05	3.12E-06	321.99	
7	M149T21	130.78	-7.03	DOWN	2.21E-05	3.23E-06	149.06	
8	M349T22	24.78	-4.63	DOWN	2.23E-05	3.23E-06	349.02	
9	M383T3	801.04	-9.65	DOWN	2.98E-05	3.55E-06	383.16	
10	M219T21_2	28.92	-4.85	DOWN	3.09E-05	3.59E-06	219.06	
11	M293T18	132.91	-7.05	DOWN	4.78E-05	4.75E-06	292.95	
12	M336T21	214.43	-7.74	DOWN	5.39E-05	5.1E-06	336.02	
13	M233T19	309.60	-8.27	DOWN	6.23E-05	5.51E-06	233.07	
14	M335T21	332.51	-8.38	DOWN	7.8E-05	6.16E-06	335.03	
15	M629T13	444.28	-8.80	DOWN	8.09E-05	6.27E-06	629.42	
16	M307T22	131.39	-7.04	DOWN	0.000102	7.15E-06	306.92	
17	M323T20_2	70.40	-6.14	DOWN	0.000121	7.83E-06	323.02	
18	M116T2_1	177.75	-7.47	DOWN	0.000124	7.94E-06	116.08	
19	M616T12	544.27	-9.09	DOWN	0.000142	8.49E-06	616.43	
20	M527T2_1	904.09	-9.82	DOWN	0.00015	8.69E-06	527.17	**5**
21	M382T2	726.51	-9.50	DOWN	0.000163	9.01E-06	382.08	
22	M251T15	38.07	-5.25	DOWN	0.000217	1.01E-05	251.05	
23	M121T21	74.30	-6.22	DOWN	0.000221	1.02E-05	121.16	
24	M630T13	692.28	-9.44	DOWN	0.000233	1.04E-05	630.44	
25	M689T2	1250.38	-10.29	DOWN	0.000241	1.05E-05	689.21	
26	M719T11	198.08	-7.63	DOWN	0.000243	1.05E-05	719.05	
27	M294T18	154.45	-7.27	DOWN	0.000251	1.06E-05	294.00	
28	M329T22	14.64	-3.87	DOWN	0.000281	1.1E-05	329.13	
29	M219T21_1	434.09	-8.76	DOWN	0.000287	1.11E-05	219.00	
30	M1055T10	2713.74	-11.41	DOWN	0.000308	1.13E-05	1054.84	
31	M365T2	431.47	-8.75	DOWN	0.000329	1.15E-05	365.16	**4**
32	M146T20	411.01	-8.68	DOWN	0.000337	1.16E-05	146.21	
33	M424T21	9.15	-3.19	DOWN	0.000362	1.18E-05	424.13	
34	M487T8	2854.33	-11.48	DOWN	0.000365	1.19E-05	487.23	
35	M253T14	25.57	-4.68	DOWN	0.000366	1.19E-05	253.02	
36	M344T20	91.34	-6.51	DOWN	0.000368	1.19E-05	344.17	
37	M453T10	365.05	-8.51	DOWN	0.000446	1.37E-05	453.19	
38	M217T21	127.50	-6.99	DOWN	0.000451	1.38E-05	217.14	
39	M163T20	125.33	-6.97	DOWN	0.000567	1.66E-05	163.08	
40	M543T2	3520.46	-11.78	DOWN	0.000588	1.71E-05	543.15	
41	M116T2_2	119.41	-6.90	DOWN	0.000778	2.18E-05	116.14	
42	M337T21	54.28	-5.76	DOWN	0.000833	2.31E-05	337.03	

43	M115T20	155.27	-7.28	DOWN	0.000964	2.61E-05	115.22	
44	M321T20_2	144.23	-7.17	DOWN	0.001127	2.97E-05	321.00	
45	M323T20_1	175.43	-7.45	DOWN	0.001634	4E-05	322.96	
46	M500T11	552.59	-9.11	DOWN	0.001769	4.25E-05	500.12	
47	M147T21	28.93	-4.85	DOWN	0.00178	4.27E-05	147.15	
48	M651T13	215.64	-7.75	DOWN	0.001838	4.37E-05	651.47	
49	M347T20	40.14	-5.33	DOWN	0.001856	4.4E-05	347.12	
50	M705T2	749.49	-9.55	DOWN	0.002051	4.75E-05	705.09	
51	M235T21	79.70	-6.32	DOWN	0.002292	5.15E-05	234.94	
52	M308T22	222.71	-7.80	DOWN	0.00234	5.23E-05	307.93	
53	M289T20	159.45	-7.32	DOWN	0.002762	5.95E-05	289.23	
54	M527T2_2	949.86	-9.89	DOWN	0.002991	6.32E-05	527.22	
55	M231T21	3.88	-1.95	DOWN	0.003109	6.5E-05	231.12	
56	M225T12	82.36	-6.36	DOWN	0.00343	6.98E-05	225.05	
57	M357T21	48.08	-5.59	DOWN	0.003571	7.19E-05	357.10	**3**
58	M429T21	3.87	-1.95	DOWN	0.003622	7.26E-05	429.24	
59	M466T20	67.69	-6.08	DOWN	0.004449	8.76E-05	465.98	
60	M343T20	306.31	-8.26	DOWN	0.005279	0.000102	343.13	
61	M415T22	56.35	-5.82	DOWN	0.006584	0.000124	415.24	
62	M539T11	237.26	-7.89	DOWN	0.007634	0.000141	539.06	
63	M305T21	1212.94	-10.24	DOWN	0.00769	0.000142	304.98	

fold change > 2, $p < 0.01$, $q < 0.01$

Table S9. List of differential secondary metabolites from root and flower IEs

No.	ID	Fold change	log2fold	Updown	*p* value	*q* value	*m/z*	Identification
1	M580T10	13.78	3.78	UP	1.77E-05	1.77E-05	580.07	
2	M595T10	18.00	4.17	UP	0.000865	0.000135	595.04	**10**
3	M579T10	21.85	4.45	UP	0.001053	0.000159	579.05	
4	M449T10_2	24.07	4.59	UP	0.005771	0.000649	449.09	
5	M450T10	33.72	5.08	UP	0.000227	5.67E-05	449.99	
6	M817T14	34.05	5.09	UP	0.003389	0.000403	817.22	
7	M584T13	37.61	5.23	UP	0.000564	9.59E-05	584.16	
8	M464T11	37.92	5.24	UP	6.3E-05	2.85E-05	464.05	
9	M449T10_1	40.79	5.35	UP	0.000628	0.000103	449.04	
10	M301T11	49.37	5.63	UP	0.000539	9.35E-05	301.18	
11	M147T14	51.48	5.69	UP	0.000507	9.04E-05	147.11	
12	M479T10	51.69	5.69	UP	4.98E-05	2.63E-05	479.00	
13	M287T10	55.03	5.78	UP	4.03E-05	2.43E-05	287.24	
14	M790T14	56.53	5.82	UP	0.000194	5.27E-05	790.25	
15	M394T14_1	62.33	5.96	UP	0.008514	0.000917	394.16	
16	M434T11	62.71	5.97	UP	0.002333	0.000298	434.04	**13**
17	M641T14	63.47	5.99	UP	2.89E-05	2.16E-05	641.32	
18	M480T10	66.02	6.04	UP	0.000202	5.38E-05	480.06	
19	M463T11	79.84	6.32	UP	0.000495	8.92E-05	463.00	
20	M642T14	99.59	6.64	UP	0.002546	0.000319	642.36	
21	M317T10	120.33	6.91	UP	0.000406	7.92E-05	317.17	
22	M271T11	123.82	6.95	UP	0.001186	0.000175	271.25	
23	M789T14	133.19	7.06	UP	1.8E-05	1.78E-05	789.28	
24	M317T13	162.14	7.34	UP	0.000107	3.77E-05	317.16	
25	M301T14	177.46	7.47	UP	0.00903	0.000966	301.22	
26	M394T14_2	187.96	7.55	UP	0.006151	0.000686	394.37	
27	M271T14	254.73	7.99	UP	1.4E-05	1.66E-05	271.25	
28	M433T11	290.51	8.18	UP	0.000709	0.000114	433.02	**11**
29	M788T14	454.77	8.83	UP	8.45E-05	3.35E-05	788.27	
30	M787T14	735.74	9.52	UP	0.000214	5.52E-05	787.27	
31	M116T2_2	6.26	-2.65	DOWN	0.001236	0.00018	116.14	
32	M116T2_1	6.49	-2.70	DOWN	8.09E-05	3.28E-05	116.08	
33	M719T11	8.18	-3.03	DOWN	7.33E-06	1.3E-05	719.05	
34	M253T14	16.25	-4.02	DOWN	0.000278	6.34E-05	253.02	
35	M329T22	34.00	-5.09	DOWN	0.00032	6.89E-05	329.13	
36	M147T21	37.65	-5.23	DOWN	0.001828	0.000247	147.15	
37	M337T21	37.88	-5.24	DOWN	0.000847	0.000133	337.03	
38	M323T20_2	44.34	-5.47	DOWN	0.000129	4.24E-05	323.02	
39	M349T22	46.51	-5.54	DOWN	3.73E-05	2.37E-05	349.02	
40	M343T20	47.83	-5.58	DOWN	0.005666	0.000638	343.13	
41	M424T21	52.04	-5.70	DOWN	0.000558	9.54E-05	424.13	
42	M251T15	53.13	-5.73	DOWN	0.000234	5.75E-05	251.05	

43	M323T20_1	59.18	-5.89	DOWN	0.001696	0.000233	322.96	
44	M415T22	60.50	-5.92	DOWN	0.00656	0.000726	415.24	
45	M357T21	60.50	-5.92	DOWN	0.003574	0.00042	357.10	**3**
46	M466T20	62.57	-5.97	DOWN	0.004451	0.000514	465.98	
47	M321T20_1	67.53	-6.08	DOWN	5.94E-10	5.66E-09	320.96	**1**
48	M429T21	68.19	-6.09	DOWN	0.001403	0.000199	429.24	
49	M225T12	69.93	-6.13	DOWN	0.003471	0.00041	225.05	
50	M347T20	72.58	-6.18	DOWN	0.001892	0.000254	347.12	
51	M145T20	77.51	-6.28	DOWN	1.49E-06	4.73E-06	145.19	
52	M382T2	88.18	-6.46	DOWN	0.00017	4.94E-05	382.08	
53	M231T21	98.58	-6.62	DOWN	0.001303	0.000188	231.11	
54	M115T20	99.46	-6.64	DOWN	0.000974	0.000149	115.22	
55	M344T20	104.67	-6.71	DOWN	0.000366	7.43E-05	344.17	
56	M146T20	109.05	-6.77	DOWN	0.000345	7.19E-05	146.21	
57	M322T20	112.68	-6.82	DOWN	1.89E-05	1.8E-05	321.99	
58	M308T22	115.07	-6.85	DOWN	0.002382	0.000302	307.93	
59	M293T18	144.63	-7.18	DOWN	4.79E-05	2.59E-05	292.95	
60	M219T21_2	163.93	-7.36	DOWN	4.1E-05	2.44E-05	219.06	
61	M163T20	166.58	-7.38	DOWN	0.000562	9.57E-05	163.08	
62	M651T13	173.82	-7.44	DOWN	0.001842	0.000248	651.47	
63	M453T10	183.47	-7.52	DOWN	0.000451	8.45E-05	453.19	
64	M336T21	185.17	-7.53	DOWN	5.4E-05	2.7E-05	336.02	
65	M365T2	187.42	-7.55	DOWN	0.000334	7.06E-05	365.16	**4**
66	M121T21	197.12	-7.62	DOWN	0.000217	5.56E-05	121.16	
67	M616T12	222.83	-7.80	DOWN	0.000143	4.48E-05	616.43	
68	M705T2	224.95	-7.81	DOWN	0.002079	0.000273	705.09	
69	M307T22	234.90	-7.88	DOWN	0.000101	3.67E-05	306.92	
70	M335T21	243.37	-7.93	DOWN	9.6E-05	3.58E-05	335.00	
71	M149T21	294.26	-8.20	DOWN	2.3E-05	1.97E-05	149.06	
72	M615T12	297.62	-8.22	DOWN	8.39E-06	1.38E-05	615.44	
73	M383T3	341.80	-8.42	DOWN	3E-05	2.19E-05	383.16	
74	M233T19	350.73	-8.45	DOWN	6.19E-05	2.84E-05	233.07	
75	M289T20	365.82	-8.51	DOWN	0.002728	0.000337	289.23	
76	M381T2	394.93	-8.63	DOWN	1.09E-05	1.53E-05	381.09	
77	M630T13	395.60	-8.63	DOWN	0.000233	5.74E-05	630.44	
78	M145T21	446.24	-8.80	DOWN	3.75E-07	1.78E-06	145.21	
79	M543T2	486.01	-8.92	DOWN	0.000592	9.89E-05	543.15	
80	M294T18	487.95	-8.93	DOWN	0.000248	5.9E-05	294.00	
81	M487T8	594.32	-9.22	DOWN	0.000368	7.45E-05	487.23	
82	M629T13	601.63	-9.23	DOWN	8.08E-05	3.27E-05	629.42	
83	M527T2_2	661.47	-9.37	DOWN	0.002996	0.000364	527.22	
84	M527T2_1	669.33	-9.39	DOWN	0.00015	4.62E-05	527.17	**5**
85	M305T21	684.56	-9.42	DOWN	0.007709	0.00084	304.98	
86	M217T21	684.70	-9.42	DOWN	0.00044	8.32E-05	217.14	

87	M219T21_1	717.71	-9.49	DOWN	0.000286	6.45E-05	219.00
88	M689T2	780.37	-9.61	DOWN	0.000241	5.83E-05	689.21
89	M235T21	795.64	-9.64	DOWN	0.0022	0.000285	234.94

fold change > 2, $p < 0.01$, $q < 0.01$
